# The Potential and Flux Landscape Theory of Ecology

**DOI:** 10.1371/journal.pone.0086746

**Published:** 2014-01-30

**Authors:** Li Xu, Feng Zhang, Kun Zhang, Erkang Wang, Jin Wang

**Affiliations:** 1 State Key Laboratory of Electroanalytical Chemistry, Changchun Institute of Applied Chemistry, Chinese Academy of Sciences, Changchun, Jilin, China; 2 College of Physics, Jilin University, Changchun, Jilin, China; 3 Department of Chemistry & Physics, State University of New York at Stony Brook, Stony Brook, New York, United States of America; University of Lleida, Spain

## Abstract

The species in ecosystems are mutually interacting and self sustainable stable for a certain period. Stability and dynamics are crucial for understanding the structure and the function of ecosystems. We developed a potential and flux landscape theory of ecosystems to address these issues. We show that the driving force of the ecological dynamics can be decomposed to the gradient of the potential landscape and the curl probability flux measuring the degree of the breaking down of the detailed balance (due to in or out flow of the energy to the ecosystems). We found that the underlying intrinsic potential landscape is a global Lyapunov function monotonically going down in time and the topology of the landscape provides a quantitative measure for the global stability of the ecosystems. We also quantified the intrinsic energy, the entropy, the free energy and constructed the non-equilibrium thermodynamics for the ecosystems. We studied several typical and important ecological systems: the predation, competition, mutualism and a realistic lynx-snowshoe hare model. Single attractor, multiple attractors and limit cycle attractors emerge from these studies. We studied the stability and robustness of the ecosystems against the perturbations in parameters and the environmental fluctuations. We also found that the kinetic paths between the multiple attractors do not follow the gradient paths of the underlying landscape and are irreversible because of the non-zero flux. This theory provides a novel way for exploring the global stability, function and the robustness of ecosystems.

## Introduction

Ecosystems are the ones in which their living and nonliving components interact with and depend on each other linking together the exchange of energy, material, information. The structure and the function of the ecosystems are determined by the interplay of both cooperation and competition [1], [2]. Ecosystems are able to regulate themselves to maintain certain stability. Therefore, the stability is one of the most fundamental and essential features of the ecological systems. The study of stability is direct relevant to the existence of every species. The stability is influenced by many factors, such as the structure within the components and the features of the environment. The studies of the stability of ecosystems are significant for uncovering the underly ecological law of species and populations [1], [2].

The ecosystems are complex and involve many kinds of interactions among the elements. The inherent interactions are often non-linear and intricate. These systems can be described by a set of nonlinear differential equations [3], [4]. These nonlinear interactions lead to complex dynamics. There have been many investigations on the stability of ecosystems [3]–[11]. Most of the works have been focused on the local linear stability analysis. The global stability of the ecological systems is still challenging in general. Furthermore, the link between the global characterization of the ecological systems and the dynamics of the elements is still not clear.

The past researchers also explored the dynamical system with the approach of Lyapunov function which was developed to investigate the global stability. The analytical Lyapunov function for predation model was first proposed by Volterra in 1931 [4]. Since then, significant efforts have been devoted to find the analytical Lyapunov function [5]–[8], [10], [11] for ecological equations. However, it is still challenging to find the Lyapunov function of the ecological models with the general nonlinear response terms, even though a few highly simplified ones were worked out [5], [8]. So far there is no general and universal approach for finding the Lypunov function. One has to work case by case. There is even no guarantee if a Lyapunov function exists for a more complex system. Furthermore the Lyapunov function and the stability of a predation model with a solution of limit cycle have hardly been discussed. Here we would like to suggest an universal and straightforward approach to explore the Lyapunov function and therefore the global stability of the general ecological systems.

In the earlier studies, people have aimed at macroscopic properties based on the deterministic models. However, both external and intrinsic fluctuations of mesoscopic and even macro scale systems are unavoidable. The environmental fluctuations can influence the ecological behaviors. The intrinsic fluctuations emerge when the size of system is finite. It is widely agreed that the analysis of the global stability is important for a full understanding of the robustness of ecological systems under fluctuations [5]–[7], [11]–[13]. As mentioned, it is challenging to explore global stability with deterministic dynamics. The probability (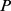) description is necessary due to the presence of fluctuations of the real systems. The probability description has the advantage of quantifying the weights in the whole population state space and therefore is global. The potential landscape 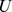 linked with the probability 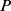 by 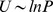 can address global properties of ecosystems, such as the global stability, function and robustness.

Here we developed a potential and flux landscape theory to explore the global stability and dynamics for ecosystems [14]–[19]. We found that the underlying intrinsic potential landscape is a global universal Lyapunov function for the ecosystem dynamics and therefore topology of the landscape provides a quantitative measure for the global stability of the ecosystems. We also found the dynamics of the ecosystems is determined by the gradient of the potential landscape and additional curl flux force from breaking down of the detailed balance.

**Figure 1:**
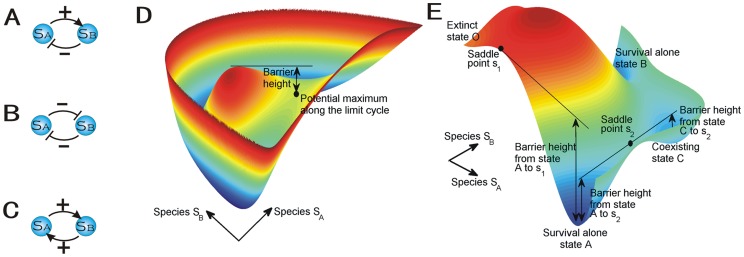
The schematic diagram for the ecological models. (A)Predation model. (B)Competition model. (C)Mutualism model. The potential landscape 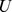 is linked with the probability 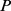 by 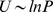 in species space. (D) Limit cycle attractor. The barrier height from the maximum inside the closed ring to the potential maximum along the ring can quantify the stability of the limit cycle attractor. (E) Multiple attractors. There are four stable states: survival alone state 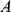 of species 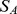, survival alone state 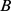 of species 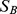, coexisting state 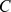, and both extinct state 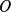. 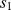 is the saddle points between the attractors 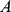 and 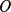 while 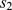 is the saddle points between the attractors 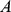 and 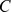. The barrier heights from the saddle points to the potential minimums of the basins can quantify the stability of each attractor.

We applied our theory to three important ecosystems: predation, competition, mutualism and a realistic lynx-snowshoe hare model. Lotka-Volterra model for two species interactions is the famous ecological model proposed by Lotka [3]and Volterra [4] respectively. Over the years, this model has attracted attentions for exploring the dynamical process of the ecology. In the ecosystems, the relationship between species can be grouped into two categories: the negative antagonism interaction(−) and the positive mutualism interaction(+). We show the different modes in Figure 1. Predation shows the relationship (+/−) which one species 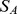 is disfavored, while the other species 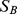 benefits in Figure 1A; Competition shows the relationship(−/−) which each species 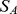 or 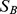 is influenced negatively by the other one in Figure 1B; Mutualism shows the relationship (+/+) which both species 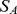 and 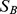 benefit from interactions of the other in Figure 1C [2], [6], [11].

For the predation, predators sustain their lives by the consumption of preys. Without the presence of prey, predators can not survive. Preys can sustain their lives and grow without predators. The presence of the preys controls the predators' growth. This forms a predator-prey(predation) system [2]–[4]. The system can have one stable state or stable limit cycle. Competition between species often occurs when they are using the same resources. Competition can promote the ecological characteristics of species differentiation and produce certain biological structure of community. The system can have multi-stable states. Mutualism means that two different species biologically interact with each other and lead to benefit each individuals. The system can also have multi-stable states. These relations above demonstrate the complexity of biological communities,their stable structures, and the ecological balance [2], [6], [11]. These models are important for population biology because of its applications to the real biological world.

Limit cycle attractor shown in Figure 1D(Mexican hat shape)and multiple attractors shown in Figure 1E emerge from these three cases: predation, competition and mutualism. Figure 1D and Figure 1E show the potential landscapes closely associated with the probability distribution for the underlying ecosystem. The lower potential landscape means the higher probability and therefore more stable states. The closed ring valley is a continuous attractor for oscillation while the discrete basins represent the stable attractors on the potential landscapes. We found the quantitative criterion for stability in terms of barrier height between basins of attraction. The barrier height from the top of the Mexican hat to the ring valley of limit cycle attractor (the potential maximum along the limit cycle) in Figure 1D or between each two attractors(from the saddle point to the minimum of each basin) in Figure 1E quantitatively determine the global stability of these ecosystems. The curl flux is essential for driving the oscillation on the valley ring and maintaining the coherence. We studied stability and robustness of ecosystems against parameters and fluctuations. We also explored how the non-equilibrium free energy links with the different phases and phase transitions of the ecosystem with respect to the changes of parameters. These explained how the stability and robustness of ecosystem change under different conditions. Quantification of pathway is important for understanding the dynamics of ecosystem. We developed the path integral method to quantify the kinetic pathway of ecosystems. We found that the paths do not follow gradient of the underlying potential landscape and are irreversible because of the non-zero flux.

## Results and Discussion

### The potential landscapes and fluxes of ecosystems: predation, competition and mutualism

We quantified the underlying potential landscape of the ecosystems through the exploration of the underlying dynamics of probability. We solved the Fokker-Planck diffusion equation describing the probability dynamics to obtain the population potential landscape 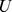 related to the steady state probability distribution 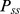 through 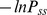 and steady state probability flux 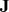. We explored the Hamilton-Jacobi equation in zero noise limit to quantify the intrinsic potential landscape 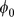 with Lyapunov properties and the associated intrinsic flux velocity. The details are in the Materials and Methods section. We will discuss how to apply the landscape theory of intrinsic potential landscape 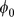, the population potential landscape 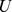 and the probability flux 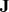 to explore the global stability and dynamics of the three ecosystem models.

**Figure 2:**
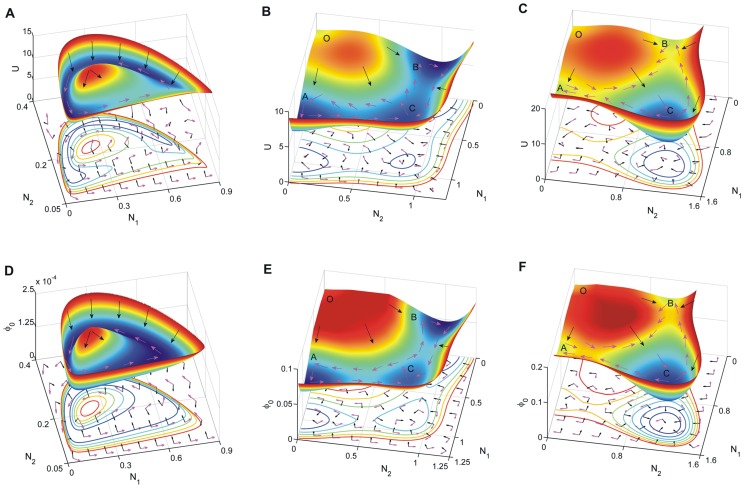
The potential landscapes for the predation, competition and mutualism models. Top row: The population potential landscape 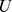 ((A) predation model. (B) competition model. (C) mutualism model.) Purple arrows represent the flux velocity(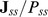) while the black arrows represent the negative gradient of population potential(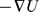). Bottom row: The potential intrinsic energy landscape 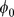. ((D) predation model. (E) competition model. (F) mutualism model.). Purple arrows represent the intrinsic flux velocity(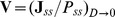) while the black arrows represent the negative gradient of intrinsic potential(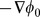).

The population potential landscape 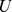 (the top row) and intrinsic potential landscape 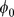 (the bottom row)for predation, competition and mutualism model are shown respectively in Figure 2. The negative gradient of the population potential landscape 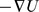 on the top row and the intrinsic potential landscape 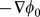 on bottom row are represented by the black arrows while the steady state probability flux 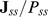 on the top row and the intrinsic flux velocity on the bottom row are represented by the purple arrows. The arrows at the bottom of each sub-figures are the projection of the direction of the according arrows. The flux with purple arrows are almost orthogonal to the negative gradient of 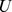 with black arrows shown on the bottom plane of Figure 2A, Figure 2B and Figure 2C. The flux velocity with purple arrows are orthogonal to the negative gradient of 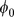 with black arrows shown on the bottom plane of Figure 2D, Figure 2E and Figure 2F.

Figure 2A and Figure 2D show the non-equilibrium population potential landscape 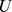 and intrinsic potential landscape 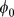 for predation model when the parameters are 
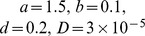
. The mathematical model and the range of the parameters are discussed in Materials and Methods section. We can see when the fluctuations characterized by the diffusion coefficient are small, the underlying potential landscape has a distinct closed irregular and inhomogeneous closed ring valley or Mexican hat like shape shown in Figure 2A. We can clearly see the population potential landscape is not uniformly distributed along the limit cycle path or the closed ring. The intrinsic potential landscape 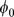 has a homogeneous closed ring valley along deterministic oscillation trajectories which has a constant value of 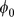 shown in Figure 2D. The non-equilibrium intrinsic potential landscape 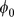 with Lyapunov properties can characterize the global topography of the oscillation landscape of predation model. This figure shows the potential is lower along the oscillation path or on the ring. The potential landscape is higher with a mountain inside the oscillation ring and outside the oscillation ring. The system is attracted to the closed oscillation ring by the landscape's gradient-potential force 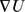 or the 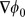. The flux driving the system maintains the periodical continuous oscillation dynamics. Both landscape and flux are necessary to characterize the non-equilibrium predation ecosystems. This oscillation shows that when the number of predators increases, more preys will be consumed. Then due to the shortage of food, the number of the predator will go down. The reduction of the predators makes the preys multiply, then the number of predators increases again for the rich preys.

Figure 2B and Figure 2E show the non-equilibrium population potential landscape 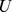 and intrinsic potential landscape 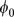 for competitive model when the parameters are 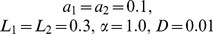. We can see the underlying population potential landscape and intrinsic potential landscape both have four distinct basins which have been discussed in the Materials and Methods section. The basins are around the four stable states. These four stable states are: survival alone state 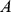 of species 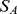, survival alone state 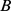 of species 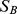, coexisting state 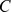, and both extinct state 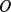. These figures show the potential landscape is relatively higher (and probability is relative lower) on the extinct state (the state 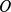) of these two species because the lower critical points 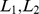 for species are small. The potential landscapes of the survival alone state 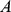 and 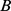 are lower(more stable) than that of the coexisting state 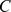. It shows that the coexisting state is less stable than the species survival alone states for they have competitive relation. The forces from negative gradient of the potential landscape are more significant away from the attractors and less significant near the basins. Therefore the system is attracted by the gradient of the landscape towards the four basins. The directions of the flux are curling among the basins.

Figure 2C and Figure 2F show the non-equilibrium population potential landscape 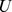 and intrinsic potential landscape 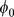 for mutualism model when the parameters are 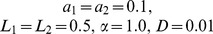. The underlying population potential landscape and intrinsic potential landscape both have distinct four basins. The basins are around the four stable states. These figures show the potential landscape is the highest (and probability is lower) on the extinct state 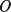 of these two species. The potential landscape of mutualism coexisting state 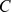 is lowest than those of species survival alone state 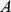 and 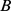, and the extinct state 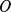. It shows that the coexisting state is more stable than the species alone state for the two species having the relationship of mutualism. The directions of the flux are curling among the four basins. And the system is also attracted by the gradient of landscape towards the four basins.

### Quantitative measure of global stability for predation

We now study the stability and robustness of the ecosystems. The stability is related to the escape time from the basins. Since the system is characterized by the basins of attractor with large weights, the easier it is to escape, the less stable the system is. We will essentially explore the average time escaping from a basin of attraction.

We define the barrier height of 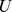 for predation model as: 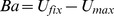. 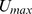 is the population potential maximum along the limit cycle attractor. 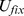 is the population potential landscape at the local maximum point inside the limit cycle. And the barrier height of 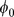 for predation model is defined as: 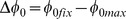. 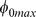 is the intrinsic potential maximum along the limit cycle attractor. 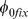 is the intrinsic potential landscape at the local maximum point inside the limit cycle. The escape time 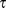 can be solved by the formula [20]: 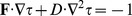. It represents the average time the system spent from one position to another position [18].

**Figure 3:**
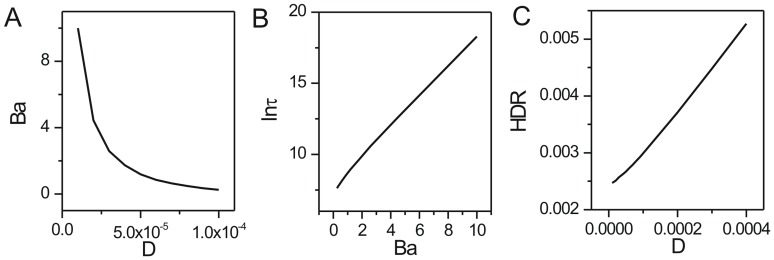
The barrier height of population landscape, escape time and dissipation rate versus the diffusion coefficient for predation model. (A) The barrier height of the population landscape 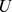 versus the diffusion coefficient 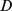. (B) The escape time versus the barrier height of population landscape. (C) The dissipation rate versus the diffusion coefficient 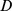.

We showed the change of population potential landscapes for increasing diffusion coefficient 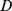 (Figure S1 in File S1). In Figure 3A, the barrier heights associated with escape time from the limit cycle attractor 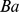 becomes higher when the diffusion coefficient characterizing the fluctuations decreases. In Figure 3B, we see a direct relationship between the escape time 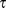 from the limit cycle attractor [17], [18] and barrier height for non-equilibrium ecosystems. As the barrier height for escape becomes higher, the escape time becomes longer. Therefore, the limit cycle attractor becomes more stable since it is harder to go from the valley ring to outside. The robustness and stability in the oscillatory predation system need small fluctuations and large barrier height. Figure 3C shows the heat dissipation rate 
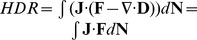
 (see the Materials and Methods section 1 for details)versus different diffusion coefficients. We can see the heat dissipation rate decreases when the diffusion coefficient decreases. This implies that when the fluctuations deduce, the associated heat dissipation rate becomes smaller. We have explored that less fluctuations lead to more robust and stable oscillation with higher barrier height. Therefore, the less dissipation can lead to less fluctuations and a more stable ecosystem with longer escape time.

**Figure 4:**
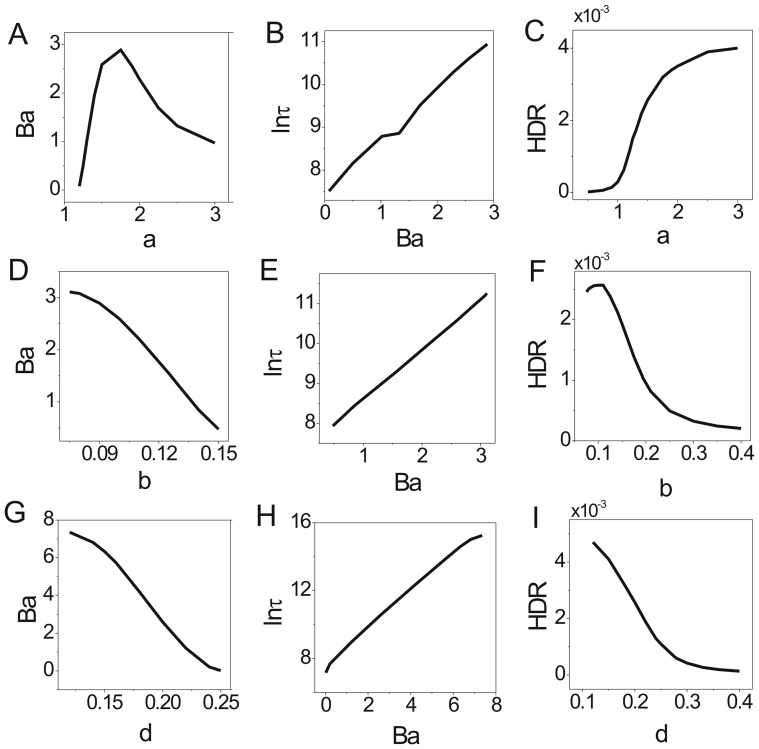
The barrier height of the population landscape, escape time and dissipation rate versus the rate parameters for predation model. (A) The barrier height of the population landscape versus 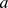. (B) The escape time versus barrier height of the population landscape for changing 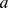. (C) The dissipation rate versus 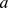. (D) The barrier height of the population landscape versus 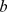. (E) The escape time versus barrier height of the population landscape for changing 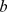. (F) The dissipation rate versus 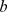. (G) The barrier height of the population landscape versus 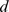. (H) The escape time versus barrier height of the population landscape for changing 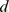. (I) The dissipation rate versus 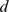.

**Figure 5:**
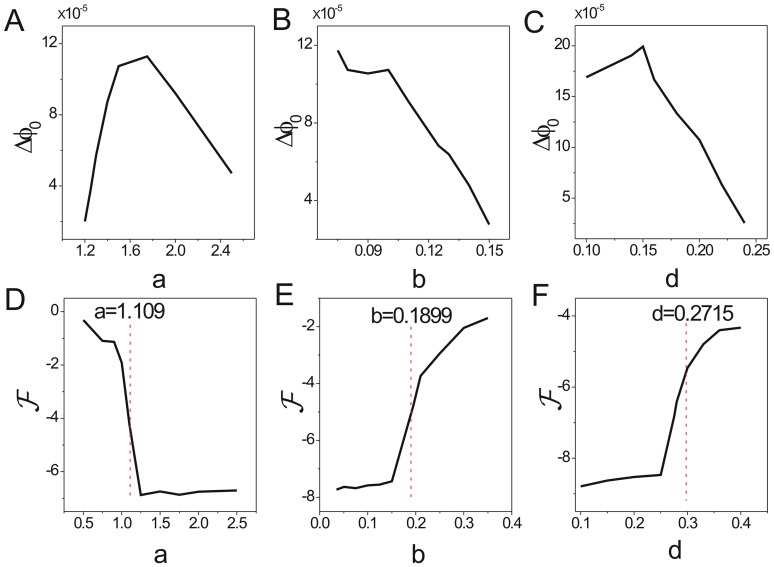
The barrier height of intrinsic potential landscape and free energy versus the rate parameters for predation model. The barrier heights of intrinsic potential landscape versus parameters 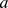 (A), 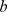 (B), 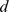 (C). The free energy versus 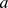 (D), 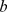 (E), 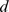 (F).

We also explored the effects of the rate parameters representing interaction strengths between species on the robustness. We showed the change of population potential landscapes for increasing parameters 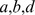 (Figure S2 in File S1, Figure S3 in File S1 and Figure S4 in File S1). We show the effects of rate parameters 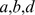 on the robustness through barrier height in Figure 4A, Figure 4D and Figure 4G; the escape time 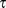 in Figure 4B, Figure 4E and Figure 4H; the dissipation rate in Figure 4C, Figure 4F and Figure 4I with 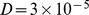. For the small parameter 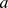, the relative death rate or the interaction strength for the prey is very small, the prey and the predator both are near their carrying capacities. The stable point is 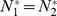, where the population of the predator is equal to the population of the prey. The system is attracted to its stable coexisting state. When the relative death rate or the interaction strength increases to a certain specific range, the system can reach a limit cycle state since it is a negative feedback system. When the parameter 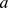 continues to increase beyond the range of limit cycle, the death rate or the interaction strength of the preys increases, the preys decrease. The lack of food leads to the reduction of the predators. So the system reaches a stable state that both predator and preys have low population. The 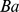 versus 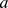 increases first, then decreases as shown in Figure 4A. Figure 5A shows barrier height 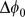 of the intrinsic potential landscape(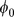) has the same tendency with that of the population landscape. It implies the system first becomes more stable, and then less stable when the parameter 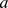 increases in the range of limit cycle. The system has an optimal stability in this range. This is due to the fact that both decreasing and increasing 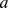 from optimal stability of the limit cycle will promote the formation of mono-stability and make the limit cycle less stable. Figure 4B shows the average escape time versus the barrier height 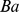. When the barrier height for escape becomes higher, the escape time becomes longer. Figure 4C shows the dissipation rate increases with the increasing parameter 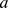. It implies that the system will consume more energy to keep order when the system stays at limit cycle oscillation state than when it stays at one stable state with the transition point 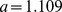. Figure 5D shows the intrinsic free energy(see the Materials and Methods section 2 for details) versus 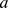. We can see clearly the non-equilibrium free energy is continuous versus parameter 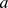, the first derivative of the free energy is discontinuous at the transition point 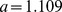 from a stable state to a limit cycle oscillation state. It is a signal of non-equilibrium thermodynamic phase transition analogous to equilibrium statistical mechanics. The non-equilibrium free energy for ecosystem can measure and predict the global phases transitions and can be use to investigate the global natural stability and robustness of the ecosystem.

When the parameter 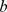 which represents the ratio of the linear growth rate of the predator to that of the prey is small, the system stays at the specific range of limit cycle state. When the parameter 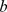 continues to increase, the growth rate of the predator increases, so the prey decreases and therefore it will lead the predator to decrease. So the system will stay at a stable state where the predator and prey both keep relative lower population. The 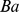 versus 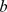 decreases shown in Figure 4D. Figure 5B shows barrier height of the intrinsic potential landscape(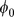) has the same tendency with the population potential landscape. This implies that the limit cycle attractor becomes less stable when the ratio of the linear growth rate of the predator increases. Figure 4E shows the average escape time versus the increasing parameter 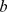 has the same tendency with the barrier height versus the increasing parameter 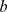. Figure 4F shows that the dissipation rate decreases with the increasing parameter 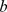. Figure 5E shows the free energy versus 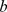. The first derivative of the non-equilibrium free energy is discontinuous at the transition point 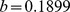 from a limit cycle oscillation state to a stable state.

The predation term 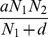 which is the response of the predator to the change in the prey density, shows the saturation effect. When the parameter 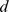 which represents the relative saturation effect rate of the prey is small, the system stays at the specific range of the limit cycle state as saturation point of predation is very low. When the parameter 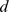 continues to increase, the response term and the death rate of the preys decreases, so the preys and the predators are in a relative lower population stable state. The barrier height 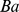 decreases as 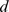 increasing shown in Figure 4G. Figure 5C shows barrier height of the intrinsic potential landscape(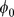) has the same tendency with the population landscape. This implies that the limit cycle attractor of this system becomes less stable when the saturation point of predation increasing. Figure 4H shows the average escape time versus the increasing parameter 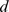 has the same tendency with the 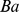 versus the increasing parameter 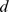. Figure 4I shows that the dissipation rate decreases with the increasing parameter 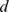. The system becomes less stable. Figure 5F shows the free energy versus 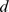. The transition point 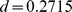 shows the system transits from a limit oscillation state to a stable state.

**Figure 6:**
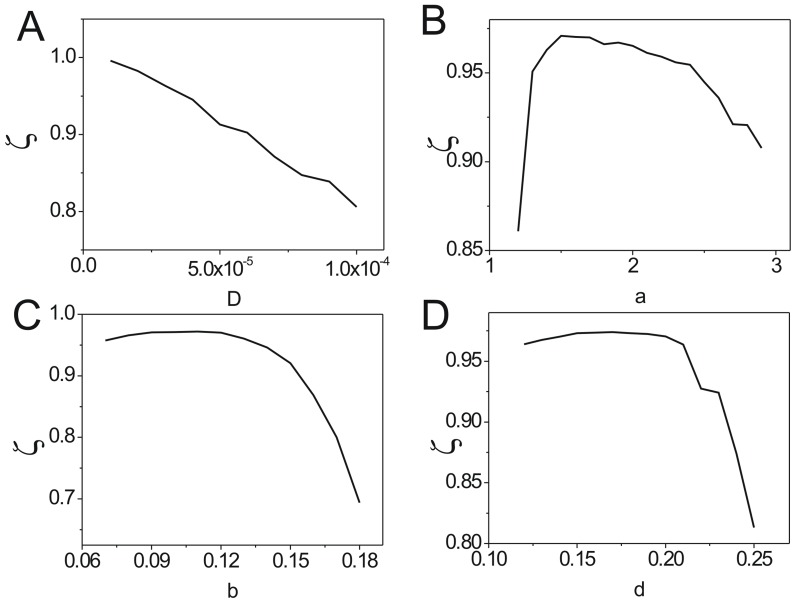
The coherence for predation model. (A)The coherence versus the diffusion coefficient. (B) The coherence versus the parameter 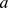. (C) The coherence versus the the parameter 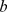. (D) The coherence versus the the parameter 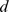.

We also can use the phase coherence to quantify the robustness and the stability of a cycle [17], [18], [21]. Coherence 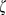 represents the degree of the regularity in time sequence of cycle motion of species variables in the ecosystem. The vector 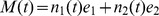 is set with 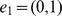 and 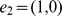 as the unit vectors, as well as 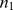 and 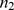 represent the population of each species at time 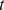. 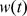 is the phase angle between 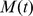 and 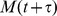. Then the 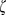 is defined as 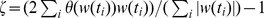 [17], [18], [21]. (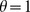 if 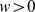, and 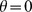 if 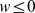) The larger value of coherence close to 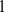 means the more periodic while the smaller value of coherence close to 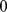 means the less periodic. In Figure 6A, 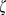 decreases when the diffusion coefficient increases. This means larger fluctuations will reduce the coherence and the robustness of the oscillations.Figure 6B, Figure 6C and Figure 6D show the 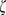 versus the parameter 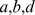. We found that they have nearly the same tendency with the barrier height versus the parameters 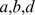 shown in Figure 4A, Figure 4D and Figure 4G. It implies that the higher barrier height lead the system to have more coherent periodic oscillation, and therefore the system is more stable.

### Quantifying the global stability of competition ecosystems

For competition and mutualism models, we introduced the effective barrier heights for simplicity. The effective barrier heights are similar to the effective resistance of parallel circuits. They can measure the effect of average barrier on a single basin. We defined the effective barrier heights for state 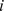 in population potential 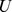 as: 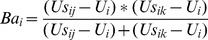 where 
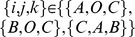
. When the system has four stable states: survival alone state 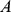 of species 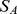, survival alone state 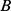 of species 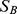, coexisting state 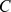, and both extinct state 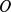. 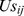 is the population landscape value of saddle point between state 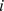 and state 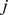. 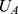, 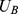, 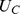 and 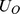 are the values of population landscape at state 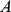, 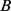, 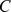 and 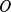. When the coexisting state 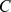 vanishes, we defined the 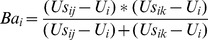 where 
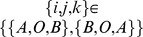
, We also defined the effective barrier heights in intrinsic potential as: 
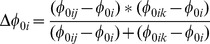
 where 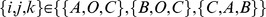 when the system has four stable states 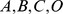. When the coexisting state 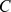 vanishes, we defined the 
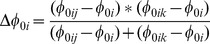
 where 
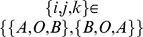
. 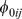 is the intrinsic landscape value of saddle point between state 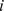 and state 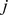. 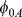, 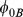, 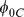 and 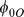 are the intrinsic potential landscape values of state 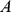, 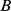, 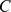 and 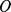.

**Figure 7:**
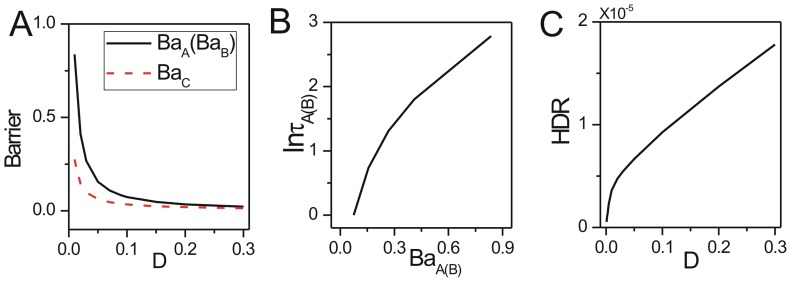
The barrier height of the population landscape, escape time and dissipation rate versus the diffusion coefficient for competition model. (A) The barrier height of the population landscape 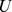 versus the diffusion coefficient 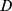. (B) The escape time versus the barrier height of the population landscape. (C) The dissipation rate versus the diffusion coefficient 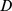.

We showed the change of population potential landscapes for increasing diffusion coefficient 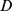 (Figure S5 in File S1). Figure 7 shows diffusion effect on the competition model. In Figure 7A, as the diffusion coefficient characterizing the fluctuations decreases, the barrier heights 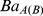 and 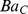 are higher when the parameters are 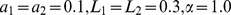. Figure 7B shows the escape time versus the barrier height 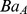. We can see the system is harder to escape from the basins of attraction as the fluctuation decreases, and the barrier height also increases. Figure 7C shows the dissipation rate for different diffusion coefficients. We can see the heat dissipation rate decreases when the diffusion coefficient decreases and the fluctuations of the systems become smaller.

**Figure 8:**
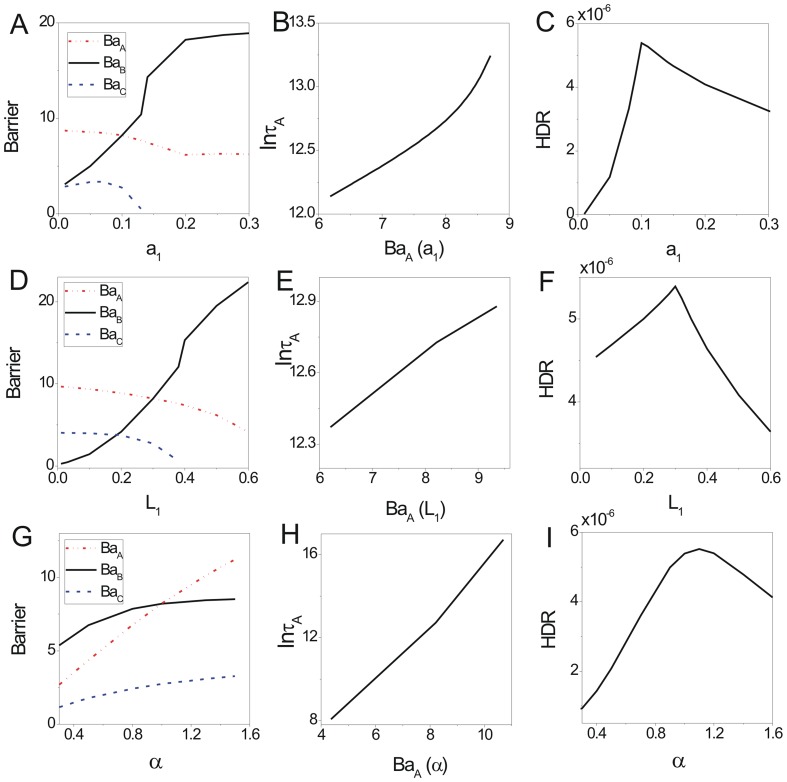
The barrier height of the population landscape, escape time and dissipation rate versus the rate parameters for competition model. (A) The barrier height of the population landscape versus 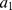. (B) The escape time versus barrier height of the population potential landscape. (C) The dissipation rate versus 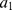. (D) The barrier height of the population potential landscape versus 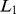. (E) The escape time versus barrier height of the population potential landscape. (F) The dissipation rate versus 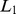. (G) The barrier heights of the population landscape versus 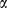. (H) The escape time versus barrier height of the population potential landscape. (I) The dissipation rate versus 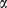.

**Figure 9:**
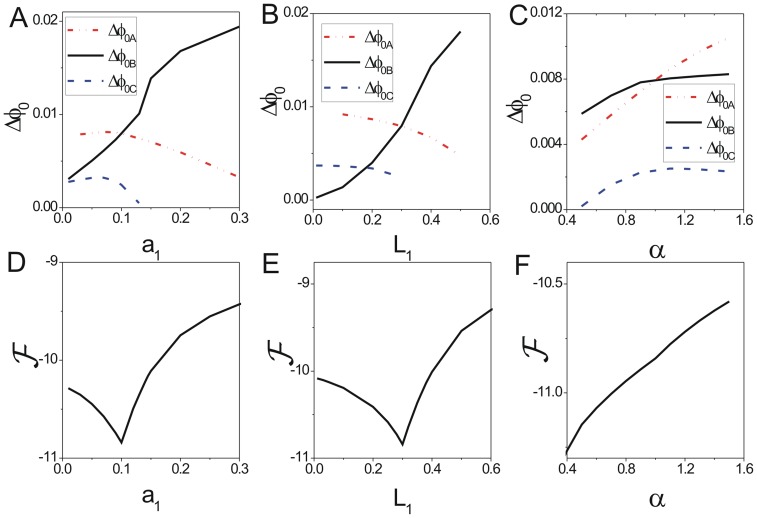
The barrier height of intrinsic potential landscape and free energy versus the rate parameters for competition model. The barrier heights of intrinsic potential landscape versus 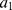 (A), 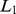 (B), 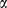 (C). The free energy versus 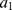 (D), 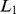 (E), 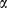 (F).

We showed the change of population potential landscapes for increasing parameters 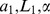 (Figure S6 in File S1, Figure S7 in File S1 and Figure S8 in File S1). Figure 8A, Figure 8B and Figure 8C show the effects of rate parameter 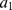 on this system at 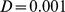. When 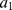, the competitiveness of 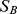 on 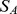 decreases, the competitiveness of species 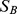 becomes weaker while that of species 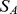 becomes stronger, so the barrier height 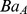 increases while 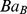 decreases. With the parameter (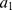) further weakened the competitiveness of the species 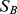, the two species can achieve their coexisting state 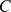. The coexisting state 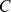 becomes more stable as the competitiveness of 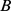 on 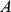 decreases shown in Figure 8A. Figure 9A shows barrier height of the intrinsic potential landscape(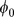) has the same tendency with that of the population landscape. This implies the two species can coexist stably as the mutual competitiveness is reduced. The logarithm of escape time 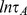 from basin 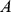 has positive correlation with barrier height 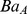 shown in Figure 8B. The escape time increases when the associated barrier height becomes higher.

Figure 8D, Figure 8E and Figure 8F show the effects of rate parameter 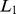 on competition system with 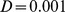. When 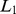 increases, the lower critical number or density of species 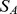 becomes larger, so the survival alone state 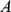 of species 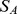 becomes less stable and shallower while the other survival alone state 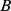 becomes more stable and deeper (The barrier height 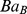 increases,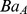 and 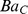 decrease as 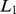 increases shown in Figure 8D. The escape time 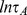 from basin 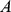 has positive correlation with 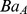 shown in Figure 8E. Figure 9B shows barrier height of the intrinsic potential landscape(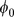) has the same tendency at those of the population landscape.), and the coexisting state 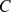 generally vanishes(The barrier height 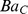 decreases). It implies that when the lower critical number or density of species 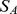 becomes larger, the species 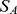 will extinct easily. The competitiveness for species 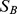 becomes larger, so it will live more stably.

Figure 8G, Figure 8H and Figure 8I show the effects of rate parameter 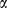 which means the relative birth rate of species 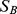 on this system with 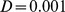. When 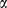 increases, the relative birth rate of species 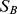 becomes larger, so the survival alone basin 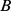 of species 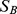 becomes more stable and deeper. The other survival alone state 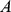 and the coexisting state 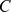 also become more stable and deeper (The barrier height 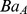 increases shown in Figure 8G and the escape time 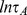 becomes longer shown in Figure 8H. They are positively correlated.Figure 9C shows the barrier height of the intrinsic potential landscape(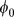) has the same tendency with that of the population landscape.) It implies that when the birth rate of 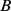 becomes larger, the species 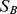 will be more stable. This can lead the coexisting state 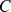 and the survival alone state 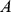 to become more stable.

Figure 8C, Figure 8F and Figure 8I show the heat dissipation rates increase then decrease when the parameter 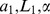 increase. The flux makes a significant contribution to the heat dissipation rate 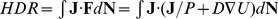. The contribution of the term 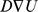 is numerically much smaller than that of term 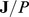 because the values of 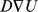 are small in less fluctuations and their directions are near orthogonality to the flux. When the parameters are given specific values: 
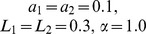
, the system stays at a symmetrical landscape topography, so the depths of the basin 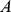 and basin 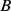 have the same value. These two basins both have large areas of dominant flux. As the three parameters increase or decrease, the system becomes less symmetrical in landscape topography, one area of the basin 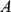 or 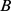 becomes more dominant. Since the area of the dominant flux becomes less, the heat dissipation rate becomes less and the system consume less energy. Figure 9D shows the phase transition from four stable states to three stable states nearby 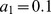. The first derivative of the non-equilibrium free energy is discontinuous at this point, which is a signal of thermodynamic phase transition. Figure 9E shows the phase transition from four stable states to three stable states nearby 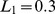. Figure 9F shows the free energy increases as 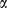 increases, and has no phase transition.

Due to competitive exclusion principle, the two species competing for the same resources are impossible to coexist in the same area. So we can see the landscape for larger 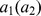 can not have the coexisting state, but it can have two stable states for living alone. The system will eventually select one stable state according to the initial condition (the slightest advantage for one species)and the fluctuations in the environment. If they coexist in the same area, there must have differences on the ecological factors, such as habitat, diet, activity time or other characteristics among the competitive species.

### Quantifying the global stability of the mutualism ecosystems

**Figure 10:**
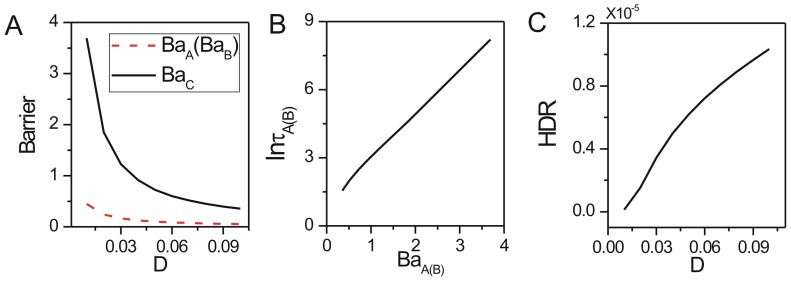
The barrier height of the population landscape, escape time and dissipation rate versus the diffusion coefficient for mutualism model. (A) The barrier height of the population landscape 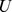 versus the diffusion coefficient 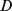. (B) The escape time versus the barrier height of the population landscape. (C) The dissipation rate versus the diffusion coefficient 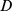.

We showed the change of population potential landscapes for increasing diffusion coefficient 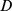 (Figure S9 in File S1). Figure 10 shows diffusion effect on the mutualism model. In Figure 10A, as the diffusion coefficient characterizing the fluctuations decreases, the barrier heights 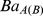 and 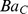 becomes higher when the parameters are 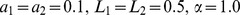. Figure 10B shows the escape time versus the barrier height 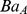. We can see clearly that the higher the barrier height is, the longer the escape time is. The system is harder to escape from the basins of attraction as the barrier height increases. Figure 10C shows the heat dissipation rate for different diffusion coefficients. We can see the dissipation or the entropy production rate decreases when the diffusion coefficient decreases.

**Figure 11:**
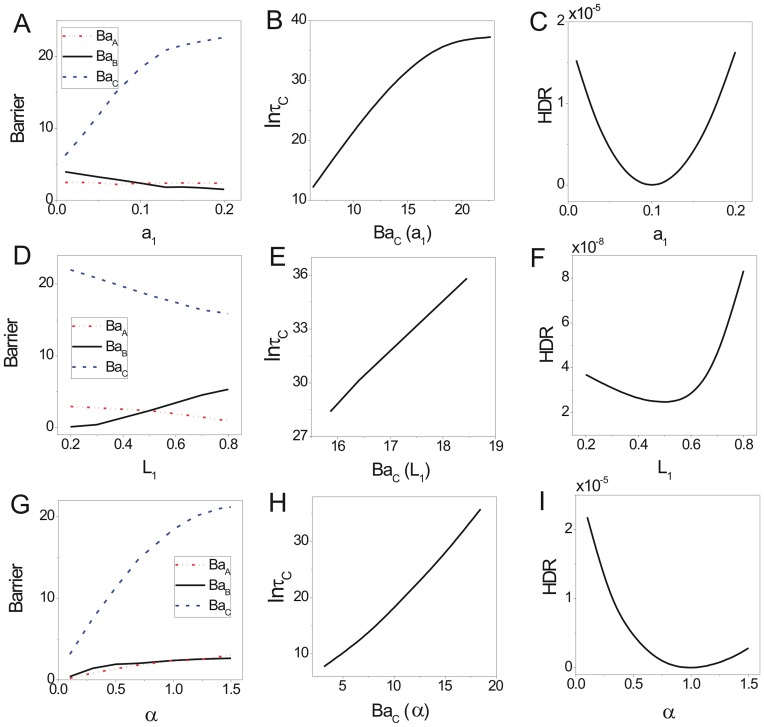
The barrier height of the population landscape, escape time and dissipation rate versus the rate parameters for mutualism model. (A) The barrier heights of the population landscape versus 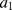. (B) The escape time versus barrier height of the population landscape for changing 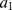. (C) The dissipation rate versus 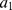. (D) The barrier height of the population landscape versus 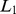. (E) The escape time versus barrier height of the population landscape for changing 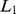. (F) The dissipation rate versus 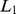. (G) The barrier height of the population landscape versus 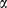. (H) The escape time versus barrier height of the population landscape for changing 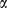. (I) The dissipation rate versus 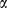.

**Figure 12:**
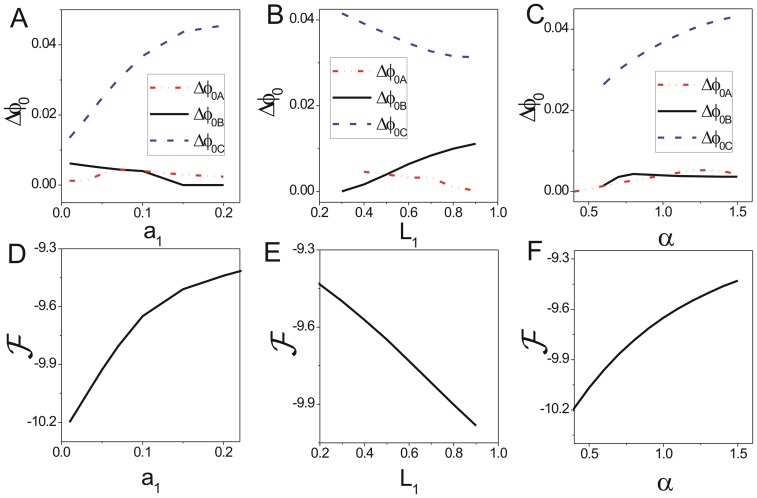
The barrier height of intrinsic potential landscape and free energy versus the rate parameters for mutualism model. The barrier heights of intrinsic potential landscape versus 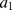 (A), 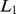 (B), 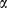 (C). The free energy versus 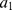 (D), 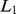 (E), 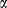 (F).

We showed the change of population potential landscapes for increasing parameters 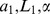 (Figure S10 in File S1, Figure S11 in File S1 and Figure S12 in File S1). Figure 11A, Figure 11B and Figure 11C show the effects of rate parameter 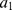 on mutualism system at 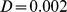. We can see when the value of 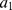, the mutualism ability is small, there are four stable states. Here we also included the lower critical number or density, so the system can go to their mutual extinction state. The coexisting state 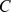 is deeper than the other two states 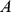 and 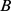 which represent the species living alone shown in Figure 11A. Figure 12A shows that the barrier height of the intrinsic potential landscape(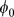) has the same tendency with that of the population landscape. This implies an obvious rule, mutual benefit can help the species to live and reproduce. As 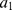 increases, the cooperative effect on species 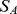 becomes stronger, the coexisting state 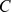 become more stable and the escape time 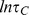 becomes longer as the barrier height 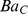 increases shown in Figure 11B. It implies that when the cooperative effect increases, the system will go to the coexisting stable state. With the increasing of the cooperative effect, the species can coexist in larger population than their respective carrying capacity.

Figure 11D, Figure 11E and Figure 11F show the effects of rate parameter 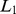 on mutualism system with 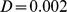. When 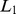 increases, the lower critical number or density of species 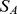 becomes larger, so the survival alone basin 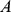 of species 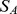 becomes less stable and shallower while the other survival alone state 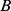 becomes more stable and deeper (The barrier height 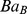 increases, 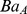 and 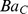 decrease as 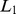 increases shown in Figure 11D. The escape time 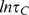 from basin 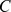 has positive correlation with 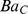 shown in Figure 11E.) When the lower critical number of species 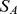 becomes larger further, the species 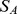 will become extinct easily. Figure 12B shows barrier height of the intrinsic potential landscape(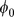) has the same tendency with that of the population landscape.

Figure 11G, Figure 11H and Figure 11I show the effects of rate parameter 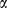 which means the relative birth rate of species 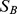 on mutualism system with 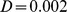. When 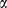 increases, the relative birth rate of species 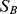 becomes larger, so the survival alone basin 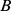 of species 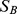 becomes more stable and deeper (The barrier height 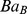 increases slightly.) The other survival alone state 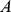 and the coexisting state 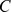 also becomes more stable and deeper shown in Figure 11G. The escape time 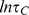 becomes longer according to the tendency of 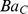 shown in Figure 11H. It implies a rule that the increase in population growth of species 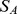 can lead to the result of greater number of species 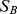, and vice versa. Figure 12C shows the barrier height of the intrinsic potential landscape(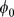) has the same tendency with that of the population landscape.

Figure 11C, Figure 11F and Figure 11I show the heat dissipation rates decrease then increase when the parameter 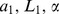 increase. When the parameters 
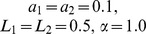
, the system stays at a symmetrical landscape topography that the depths of the basin 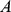 and basin 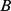 have the same value. In this case, these two basins both have less contribution to the 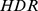 since the flux in coexisting basin 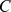 is much larger than those in basin 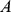 or 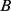. As the three parameters increase or decrease, one area of the basin 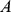 or 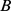 becomes generally more dominant than that of the symmetric landscape topography in addition to the area of basin 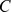. Since the area of dominant flux expands, the heat dissipation rate becomes larger and the system needs to consume more energy.

Figure 12D and Figure 12F shows that the free energy increases as 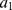 and 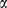 increase.Figure 12E shows the free energy increases as 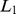 decreases. We can see there is no discontinuous changes in the first derivative of the free energy since there is no phase transition phenomenon.

### Quantifying the kinetic paths for the non-equilibrium ecosystems

**Figure 13:**
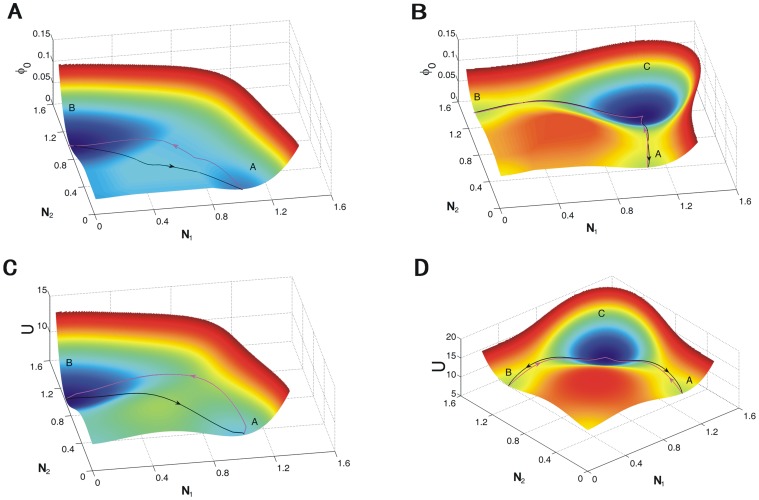
The paths for competition model and mutualism model. (A) The paths on the intrinsic potential landscape 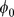 for competition model. (B) The paths on the intrinsic potential landscape 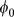 for mutualism model. (C) The paths on the population potential landscape 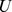 for competition model. (D) The paths on the population potential landscape 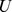 for mutualism model.

We showed the kinetic paths for the ecosystems on the landscapes(see the Materials and Methods section 3 for details). Figure 13A and Figure 13B show the kinetic paths on the intrinsic potential landscape 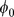 for competition model (We use the parameters 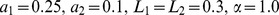 which has no coexisting state 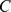 in order to see the paths clearly here.) and for mutualism (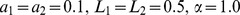) model respectively. Figure 13C and Figure 13D show the kinetic paths on the population potential landscape 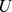 for competition model and mutualism model with 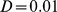. We can see the paths by purple line from state 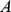 and state 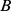 are quite different from the paths by black line from state 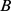 to 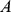 in Figure 13A and Figure 13C. The paths from 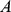 to 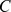 (from 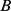 to 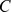) by purple line and the paths from 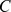 to 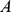 (from 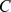 to 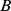)by black line are shown in Figure 13B and Figure 13D. We can see these pathways do not follow the gradient paths on both the intrinsic potential landscape and the population potential landscape due to the non-zero flux. The pathways do not necessarily pass the saddle point which is not similar with the equilibrium system. The forward and backward kinetic paths are irreversible which provides a clear signature of the non-equilibrium system. We can explore the detailed dynamical mechanism of ecosystems by quantifying the kinetic paths.

### Quantifying the global stability and dynamics for Canada lynx and snowshoe hare population cycle

The lynx-snowshoe hare cycles show the typical predator-prey behaviors which many researches have tried to do realistic modeling on [2], [22], [23]. The modeling equations describing the behavior of lynx-snowshoe hare cycles were proposed and there exists limit cycles under much wider scope of parameters with consideration of the Holling-type II functional responses for lynx and Holling-type III functional responses for general predators, such as coyote and great horned owl in the boreal forest [2], [22]–[24]. Here we use a model of lynx-snowshoe hare cycles shown as [22]–[24]: 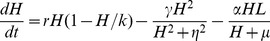, 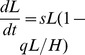, where 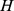 and 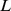 are the population density of hares and lynx. 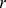 is the growth rate of hares, 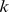 is the carrying capacity, 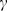 is the maximum killing rate of general predation, 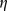 is the half-saturation constant for general predation [22]–[24]. 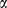 is the maximum killing rate of lynx, 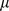 is the half-saturation constant for lynx' predation [22]–[24].

**Figure 14:**
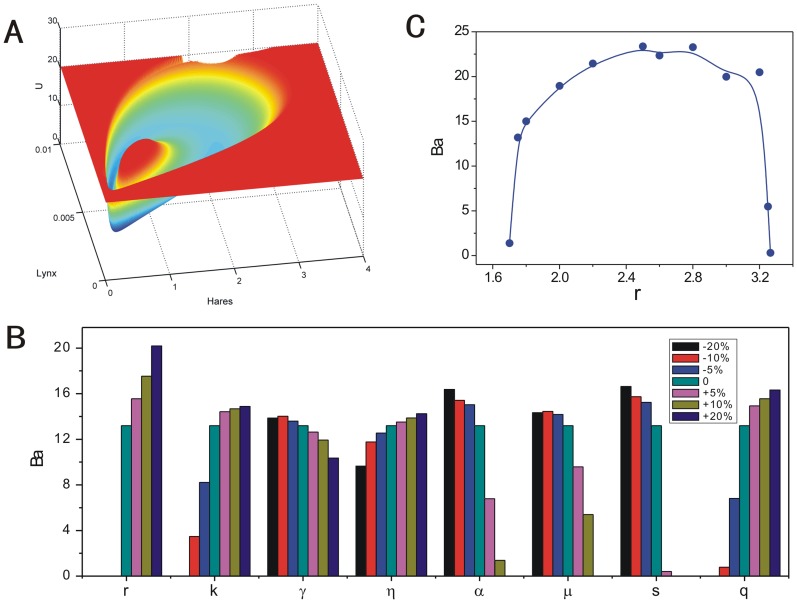
The potential landscape, barrier height of the population landscape and the sensitivity of parameters for lynx-snowshoe hare model. (A) The population potential landscape for lynx-snowshoe hare model. (B) The barrier heights versus changing parameters. The basic set of the parameters are: 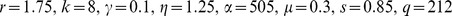. (C) The barrier heights versus the hares' rate of population growth.

We can not ensure which oscillation is more robust by only the analysis of the local stability of differential equations. Therefore, we have explored the stochastic dynamics of this model and quantified the global stability using our landscape and flux theory. Figure 14A shows the population potential landscape with the basic set of parameters [22]. Figure 14B shows the sensitivity of each parameter with respect to the landscape topography through the barrier heights which can be used to quantify the robustness and global stability of the lynx-snowshoe hare cycles. The parameters decrease by 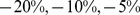 and increase by 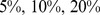 of their own values. The color bars which are not displayed for each parameter, such as the disappearances of black and red bars for parameter 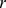, indicate the disappearance of the periodical oscillations for that value of certain parameters. We can see the changing of 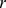 have significant impact on the robustness of the oscillation while the changes of the parameter 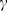 and 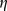 have less significant impacts on the robustness of the oscillation. We showed a more detailed analysis of the robustness for changing parameter 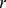 in Figure 14C. There exists an optimal value 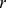 in Figure 14C. The optimal value of 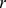 leads to a more robust and stable ecological oscillation of hares and lynx. The barrier heights from the potential landscape topography can be used to quantify the global stability and the robustness of the oscillations. We can explore which set of parameters will lead the ecosystem to have more robust oscillation with higher barrier heights. This will help to design strategy to preserve the ecosystems.

## Conclusion

Stability and dynamics are crucial for understanding the structure and function for ecology. Ecological stability is commonly defined as Lyapunov stability to describe the global stable behavior of ecosystem upon perturbations.Unfortunately, in general, Lyapunov stability cannot be assessed because explicit Lyapunov function can hardly be found. In this study we have illustrated a general method to explore the Lyapunov global stability of the ecosystem through the quantification of the underlying intrinsic landscape. It can be used to explore more general complex ecosystem where the situation can only be studied case by case before. We found the dynamics of the ecosystems is not only determined by the gradient of the potential landscape but also by an additional curl flux force from breaking down the detailed balance. This provides a new way to explore the general dynamics in non-equilibrium regime for ecosystems.

We considered three important ecological systems with the interactions between two species: the predation model, the competition model, the mutualism model and a realistic lynx-snowshoe hare model. Multiple attractors and limit cycle attractor with a distinct Mexican hat shape emerge from these cases. We found the quantitative measure for global stability through barrier height. The non-equilibrium free energy can reflect the global phases of the underlying ecosystems and the transition regions between the global phases. We quantified the pathways of ecosystem which do not follow the gradient path on the landscape and are irreversible. We quantified the kinetic speed from one stable state basin to another of the ecosystems and linked with the underlying landscape topography through the barrier height between the basins of attractions.

For ecosystems, the stability is directly related to the survivals of every species. We showed the effects of parameters representing the interactions among species on the global natures such as landscape topography represented by barrier height, kinetics speed represented by escape time and the thermodynamic dissipation by the entropy production or heat dissipation rate in these ecosystems. Therefore we can quantify the change of the stability by increasing or decreasing the interaction parameters, respectively. These results can help us to design more stable ecosystems.

The ecosystem dynamics shares some common global features as the biochemical systems such as gene-gene regulations, in terms of the underlying landscapes, the global stability and dynamics, kinetic rate and pathways. There are also significant differences between these two types of systems. First of all, the ecosystems and gene-gene regulation systems are at completely different level, one is on the population species and the other is within the cell. Second, their components have different sensitivity against changes. On the one hand, it is relatively easier to mutate the genes and harder to mutate the species. On the other hand, it is relatively easier to change the link between the species due to the sensitivity of ecosystems to the environment rather than the gene-gene regulations. Third, the ecosystems depend more sensitively to the outside input than the gene-gene regulations.

It is worth mentioning here that the landscape ecology is an emerging subfield in ecology. The landscape ecology models concentrate on spatial heterogeneity with space probabilistic methods [25], [26]. Although these methods and our theory all focus on the dynamical evolution in probability, we concentrate on the probability landscape and flux in the population space rather than in the geographical space as in the landscape ecology models.

The realistic models are usually more complex. The models often have more parameters controlling the dynamical behaviors of the ecosystem. We can apply our potential and flux landscape framework to quantify the stability of those systems irrespect to how complicated the underlying processes dictated by the differential equations may be. Furthermore, we can explore the set of parameters for more stable system by the underlying landscape topography quantified by the barrier heights. Thus the parameter-control can be realized to achieve the ecological stability and ecological decision makings.

For a mutualism model like honeybee and flowering plants, the local analysis of the differential equations can only give the existence of the stable states and local stability but not the global robustness of the stable states. The honeybee and flowering plants can coexist in a wide range of their dynamical parameters. Our potential and flux landscape framework can give in which conditions the honeybee and flowering plants can more robustly coexist. We can also quantify the changes of the robustness of ecosystem through the changes in controlling parameters for the underlying landscape topography.

We can also use our potential and flux landscape theory to explore the interactions of multi-species as a multi-node network [15], [27]. The analysis of sensitivity from the potential landscape can explore the effects of specific parameters or wirings to robustness and stability of the ecosystem. We can identify the key species or wirings that are responsible for the global stability and function of the whole ecosystem through the potential landscape topology.

The stability of ecosystems is a challenging issue. We have focused on our discussion with low dimensional models in this study. In realistic ecosystems, the global stability can seldomly be quantified by just exploring the stability with a few differential equations. The stability must be viewed as a multi-dimensional problem. It is determined by many internal and external degrees of freedom including population density, temperature, space, age,other competitors and environmental factors. A population dynamics P system (PDP) model as a tool for modeling and simulating complex ecosystems was proposed with a high computational modularity, efficiency, and the parallelism [28], [29]. We will need to go further to explore the even more complex ecosystems with the consideration of other factors.

The methods developed in this study can be applied to more complicated and realistic ecosystems to understand the global stability, function and robustness.

## Materials and Methods

### Quantifying the non-equilibrium population and intrinsic potential, flux, Lyapunov function and non-equilibrium thermodynamics for general ecological systems with finite fluctuations

The ecological systems are not in isolations since there are often intrinsic and extrinsic environmental noises around them [30]. So the dynamics can be described as follows [20], [31]: 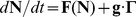. The vector 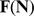 is the deterministic force that drives the system, where 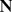 is the vector of dynamical variables, with each component of which representing different species in the ecosystems. 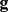 is the noise force from the fluctuations. The statistical nature of the noise can often be assumed as Gaussian white noise: 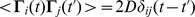 (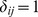, if 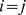; 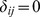 if 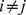) and 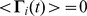. 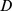 is the diffusion coefficient representing the level of noise strength while 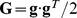 is the scaled diffusion matrix described the anisotropy phenomenon. We set 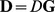.

Under fluctuations, the individual trajectories are stochastic and do not have the predictive power, we should focus on the evolution of probability distributions rather than evolution of individual trajectories. We can explore the corresponding stochastic dynamics governed by the Fokker-Planck diffusion equation [20], [31] for the temporal evolution of the probability distribution 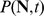:
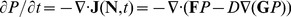
(1)This represents a conservation law of probability (local change is due to net flux in or out). The diffusion equation essentially describes the probability of finding the state 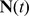 in the state space which is driven by the driving force 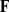. The probability flux vector 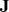 of the system in population space 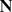 is defined as: 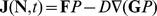.

In steady state, 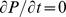, therefore 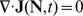. 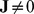 means the detailed balance is broken and the ecosystem is in non-equilibrium state. The divergence-free property implies the flux 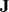 is rotational. From the definition of 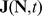, one can write the force decomposition: 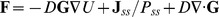. In this way, the driving force 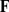 can be decomposed into the gradient of the potential 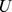, the divergence of the diffusion coefficient and the curl probability flux. Therefore the global nature can be determined by the non-equilibrium population potential 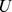 characterizing the probability landscape of the whole population space, the non-equilibrium local dynamics and their global properties can be determined by both the gradient of the potential landscape and the rotational curl flux [17]. This is in contrast with the zero flux case for detailed balance of equilibrium systems, where the global nature is determined by the equilibrium potential, and the dynamics is determined by the gradient of the equilibrium potential.

The steady state probability distribution under the fluctuation and the non-equilibrium population potential of ecosystems is quantitatively linked by: 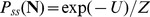, where 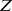 is the partition function for non-equilibrium ecosystems defined as 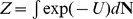. Then the entropy of the non-equilibrium ecosystem is given as [14], [18], [32]–[34]: 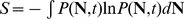, and the energy of the non-equilibrium ecosystem is given as: 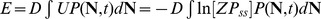. Therefore, the free energy 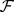 of the non-equilibrium ecosystem can be defined as:
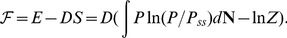
(2)The free energy is a combination of non-equilibrium energy and entropy, suggesting the first law of non-equilibrium thermodynamics for ecosystems. The free energy decreases in time monotonically until it reaches its minimum value, 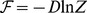 [14], [18], [32]–[34]. The free energy of these non-equilibrium ecosystems never increases, suggesting the second law of non-equilibrium thermodynamics for ecosystems. We can use the free energy to explore the global stability of non-equilibrium ecosystems with finite fluctuations from the environments or intrinsic sources.

We also investigated the derivatives of the entropy in the time evolution [14], [18], [32]–[34]. It can be decomposed as follows: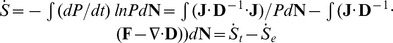 The first term is entropy production rate 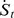, having the physical significance of the total entropy change of system and environment. It is always non-negative corresponding to thermodynamic second law. The last term is the heat dissipation term 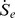 from the environments, and it can be either positive or negative [14], [18], [32]–[35]. The entropy of the ecosystems is not always increasing, but the free energy of the ecosystems reduces itself to a minimum in time. It can be used as an optimal principles to explore the topologies and the design of ecosystems.

### Quantifying the non-equilibrium intrinsic potential and flux, Lyapunov function and global stability for general ecosystems with zero fluctuations: deterministic ecosystems

The essence of the stability-problem is to analyze that how a ecosystem returns to the original state under an initial perturbation. Lyapunov function is traditional used to quantify the global stability of dynamical ecosystems. But the population potential 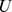 we have obtained does not necessarily have the property of the Lyapunov function. [14]–[18], [ 32]–[34], [ 36]–[40]. Some analytical solutions of the Lyapunov function can be found for special simplified ecosystem models [5], and they can only be analyzed case by case. For complex real ecosystems, it is still challenging to find a proper Lyapunov function. We show here a general approach to obtain the Lyapunov functions for dynamical ecosystems.

The underlying intrinsic potential for the dynamic system can be obtained under the weak noise limit 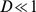. Therefore, we can expand the population potential 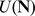 according to the fluctuation strength 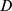, and the associate probability 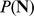 is as the following form [18], [35], [36], [38], [41]:
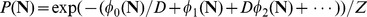
(3)where 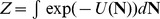. We can substitute the expanded equation to the Fokker-Plank diffusion equation.Therefore we have the 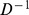 order expansion of the Fokker-Plank diffusion equation: 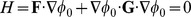. The equation 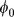 followed is called Hamilton - Jacobi equation(HJE). We can solve the Hamilton-Jacobi equation which is zero-fluctuation limit of the Fokker-Planck equation to get the intrinsic potential 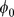 by a numerical method - level set method using the Mitchell's level-set toolbox [42].

Then we can study the deterministic equation for 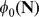 [18], [41]. We can explore the time evolution of the 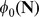 in the zero fluctuation (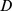) limit:
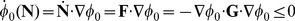
(4)Because 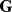 is positive definite, the value of 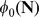 monotonously decreases under the deterministic evolution equation (zero fluctuation limit). Therefore 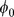 is a Lyapunov function, which can be used to quantify the global stability of the ecosystems. In addition, 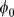 is related to the population potential 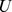 under weak noise limit by 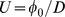. So 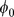 is linked with the steady state probability which can quantify the global properties for ecosystem.

For the deterministic systems, we can take the zero-fluctuation limit and follow the procedures described above to obtain the non-equilibrium energy, entropy and free energy as well as the corresponding non-equilibrium thermodynamics. Furthermore, we can also recover the force decomposition for the deterministic ecosystems in zero fluctuation limit as gradient of non-equilibrium intrinsic potential 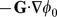 and steady state intrinsic divergent free curl flux 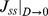: 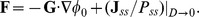. From the Hamilton-Jacobian equation above, we see that 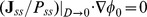. This means the gradient of the non-equilibrium intrinsic potential 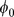 is perpendicular to the intrinsic flux under the zero-fluctuation limit [18], [36], [38].

### Quantifying the paths of ecosystems

Quantification of the pathways of the ecosystems gives us an opportunity to explore the ecological dynamical process. We assume the path probability starts from initial configuration 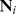 at 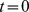, and end at the final configuration of 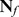 at time 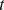. The path integral formula is [43], [44]:
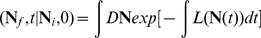
(5)where 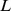 is the Lagrangian of the ecosystem [43], [44]:
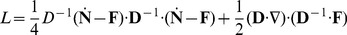
(6)The path integral over 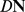 represents the sum over all possible paths connecting 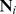 at time 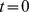 to 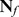 at time 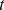. The term 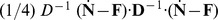 gives the weight contribution from specific path from the underlying Gaussian noise. The term 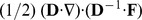 gives the contribution from the Jacobian variable transformation from the Gaussian noise to the path. The 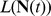 represents the weight for each path. The probability of ecosystem dynamics from initial configurations 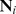 to the final state 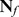 is equal to the sum of all possible paths with different weights. Not every dynamical path contributes to the same weight. We can identify the dominant paths which give the most contribution to the weight. This approximation is based on the fact the weight is exponentially weighted. The sub-leading contributions are exponentially small. Therefore dominant paths which give the most contribution to the weights can emerge. We explored the dominant kinetic paths from one state to another for ecosystems in this study.

### The ecological dynamical models

The ecosystems can be described by a set of nonlinear ordinary differential equations for two species interactions. We will add some restrictions on the models to enable them to be more reasonable and closer to the real situation [2], [45], such as avoidance of exponential growth and existence of lower critical bound for each species.

(1)Predation Model

The general Holling type II responses for the prey to account the nonlinearity in interactions can be added in a predation model proposed by Murray [2]:
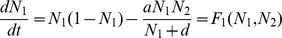


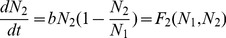
(7)where 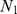 is the normalized population of prey while 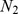 is the normalized population of predator.The parameter 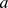 is the relative death rate or the interaction strength for the prey. The parameter 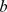 is the ratio of the linear birth rate of the predator to that of the prey. The parameter 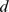 is the relative saturation rate of the prey. The system has two general saddle points: one is 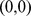 representing that none of the species exists. The second one 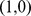 represents the prey at their carrying capacity in the absence of predators [2]. The second point is stable along the 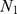 population axis and unstable along the 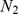 population axis. There is also a critical point which is the unstable center of the limit cycle or the stable point in different parameter ranges. The system has a stable limit cycle oscillation when we set 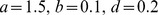.

(2) Competition Model

The realistic competitive model should have with a lower critical bound, which means the creatures would perish once the size of the population is below this threshold. The model is shown as follows [45]:
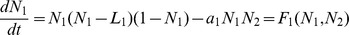


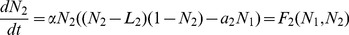
(8)where 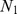 and 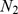 are the normalized populations of the two competitive species 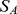 and 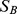. 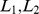 are the lower critical bounds for species 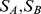, respectively. The ranges of 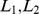 are from 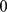 to 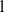. 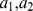 are the competitive ability for species 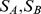, respectively. 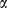 is the relative rate of natural increase for species 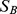 [45].

We have explored the phase analysis of the system. The two kinds of population which are both at zero mean the two species both are at extinct state 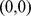 (Marked as 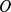) of the system. This is because the two groups both have lower critical density. When there are no competitors for each species, the states: (1,0) (Marked as 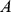, which means the species 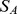 exists alone.) and (0,1) (Marked as 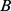, which means the species 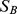 exists alone.) that exist in isolation are locally stable. Besides the above three states, when the values of 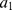 and 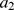 meet certain conditions, the system can have another local stable state which corresponds to the coexistence of the two species (Marked as 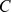). Here, we set 
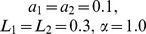
 since it has these four states.

(3) Mutualism Model

We will consider the two mutualism species both having lower critical bound. This realistic mutualism model can be described as [45]:
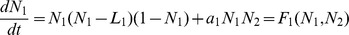


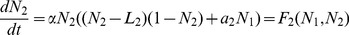
(9)where 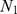 and 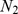 are the normalized populations of the two mutualism species 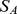 and 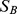. 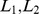 are lower critical points for species 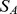 and 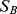, respectively. The ranges of 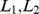 are from 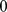 to 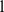. 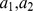 are the mutualism ability for species 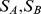, respectively. 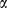 is the relative rate of natural increasing for species 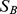 [45].

We have explored the phase analysis of this system. The two kinds of population which are both at zero mean the trivial solution 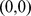 (Marked as 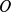) of the system. This is because the two groups having a lower critical density. When there is no mutual helper for each species, the states: (1,0) (Marked as 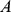, which means the species 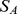 exists alone.) and (0,1) (Marked as 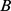,which means the species 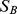 exists alone.) that exist in isolation are locally stable. Besides the above three points, the system has another local stable point which corresponds to the coexistence of the two species (Marked as 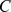). Here, we set 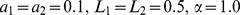 for the system has these four states completely.

## Supporting Information

File S1Supporting figures. **Figure S1.** The population potential landscapes for increasing diffusion coefficient 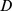 of predation model. **Figure S2.** The population potential landscapes for increasing parameter 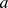 of predation model.**Figure S3.** The population potential landscapes for increasing parameter 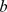 of predation model. **Figure S4.** The population potential landscapes for increasing parameter 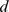 of predation model. **Figure S5.** The population potential landscapes for increasing diffusion coefficient 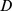 of competition model. **Figure S6.** The population potential landscapes for increasing parameter 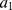 of competition model. **Figure S7.** The population potential landscapes for increasing parameter 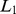 of competition model. **Figure S8.** The population potential landscapes for increasing parameter 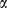 of competition model. **Figure S9.** The population potential landscapes for increasing diffusion coefficient 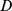 of mutualism model. **Figure S10.** The population potential landscapes for increasing parameter 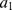 of mutualism model. **Figure S11.** The population potential landscapes for increasing parameter 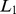 of mutualism model. **Figure S12.** The population potential landscapes for increasing parameter 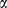 of mutualism model.(DOC)
