# Supplementary material for: The Potential and Flux Landscape Theory of Ecology
Source: PLoS One. 2014 Jan 30;9(1):e86746. doi: 10.1371/journal.pone.0086746 (PMC3907570; doi:10.1371/journal.pone.0086746)

**Supporting Information**

**Figure S1** The population potential landscapes for increasing diffusion coefficient D of predation model.


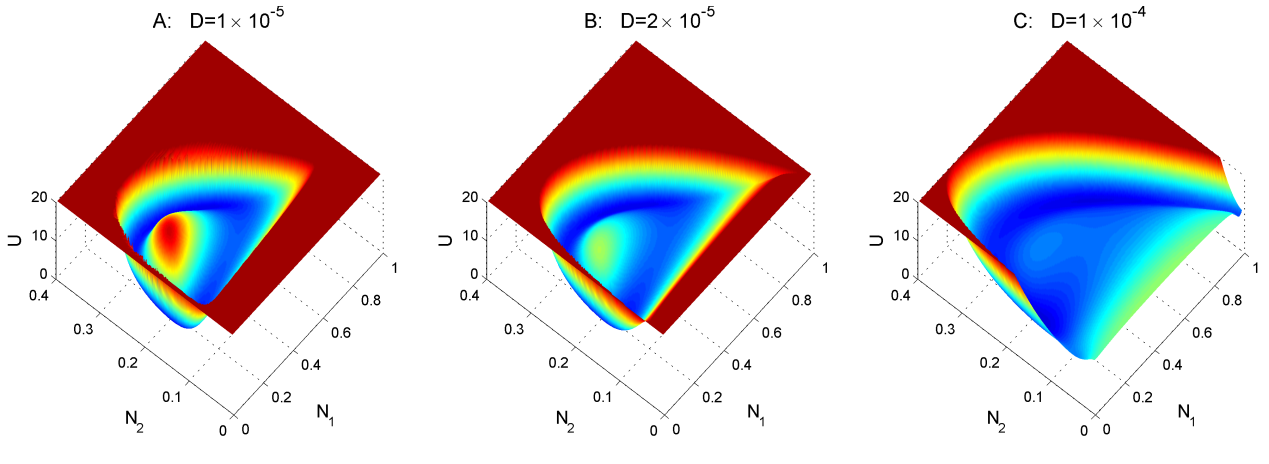


**Figure S2** The population potential landscapes for increasing parameter a of predation model.


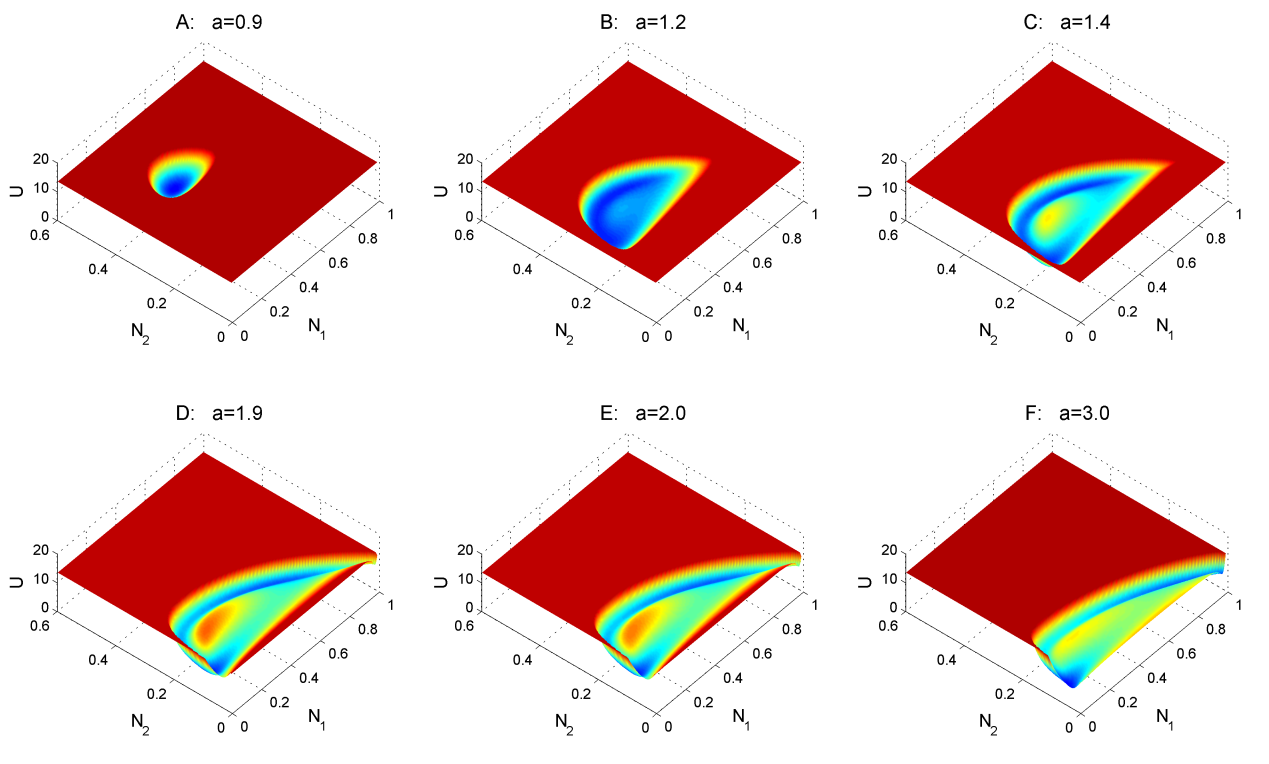


**Figure S3** The population potential landscapes for increasing parameter b of predation model.


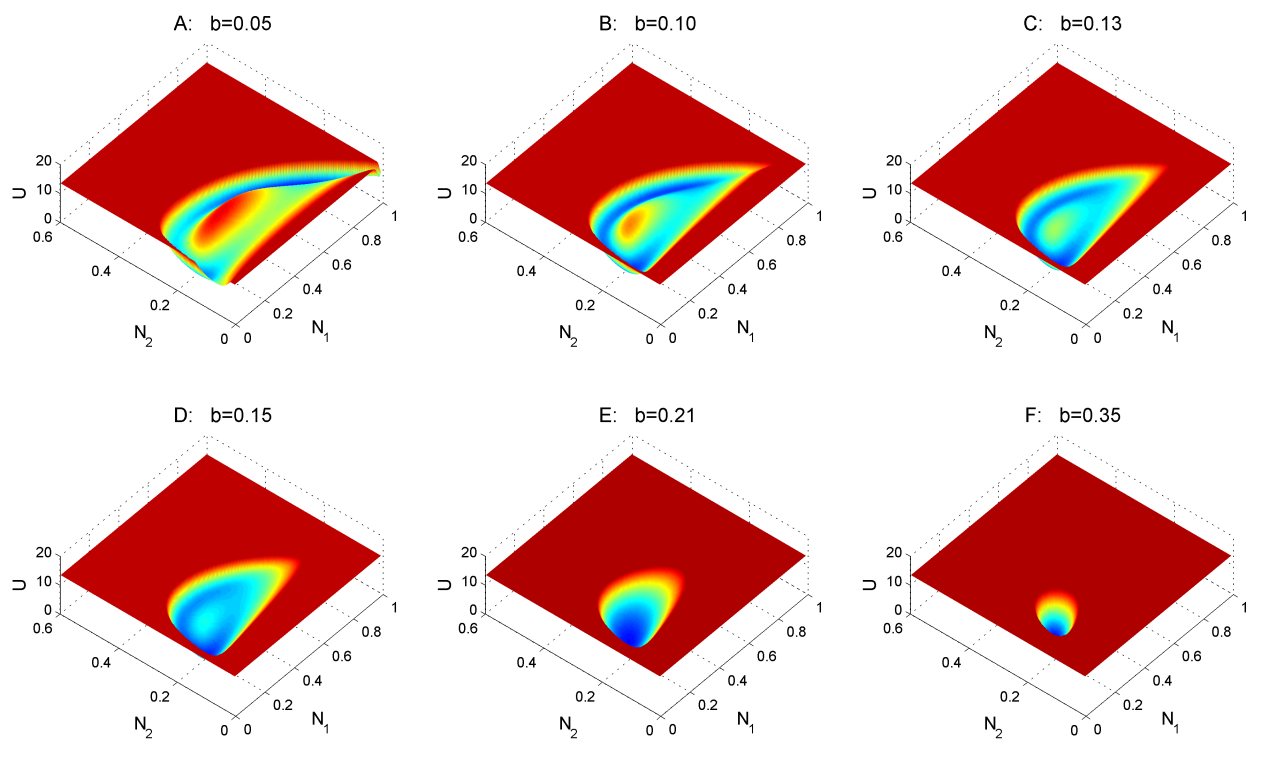


**Figure S4** The population potential landscapes for increasing parameter d of predation model.


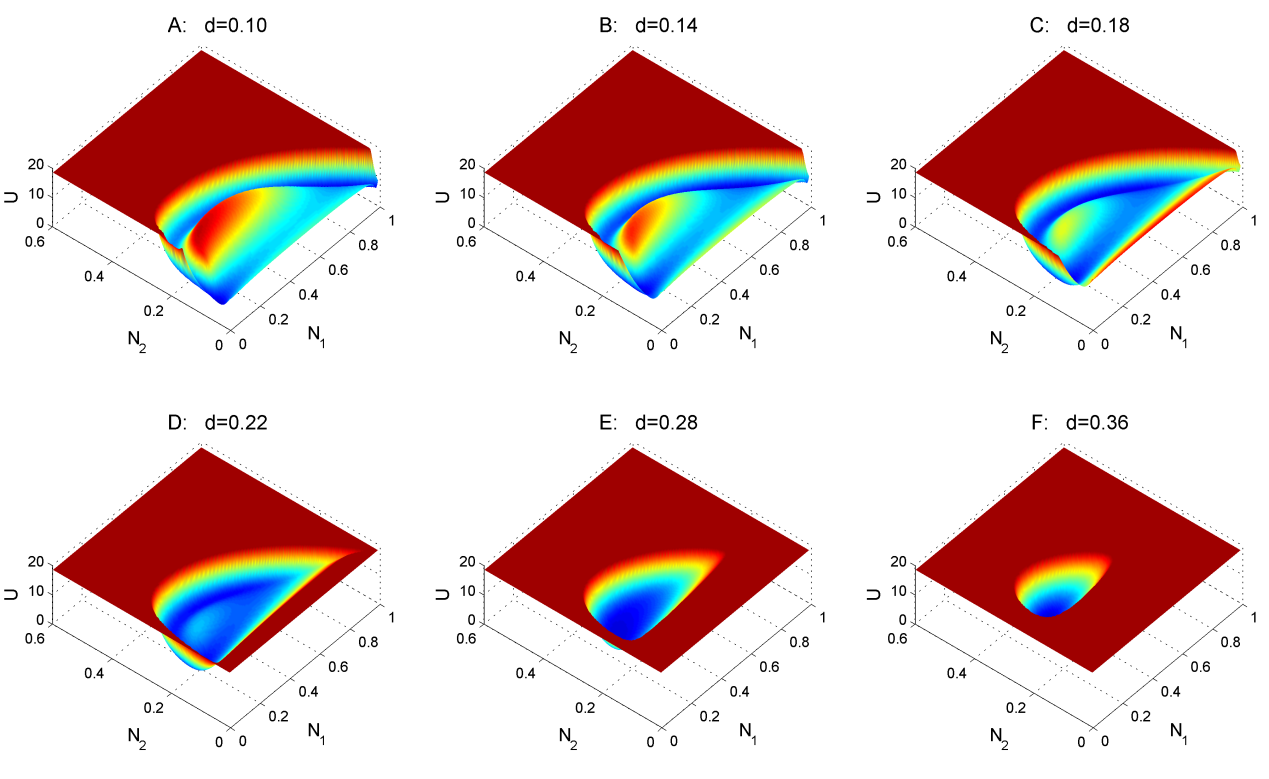


**Figure S5** The population potential landscapes for increasing diffusion coefficient D of competition model.


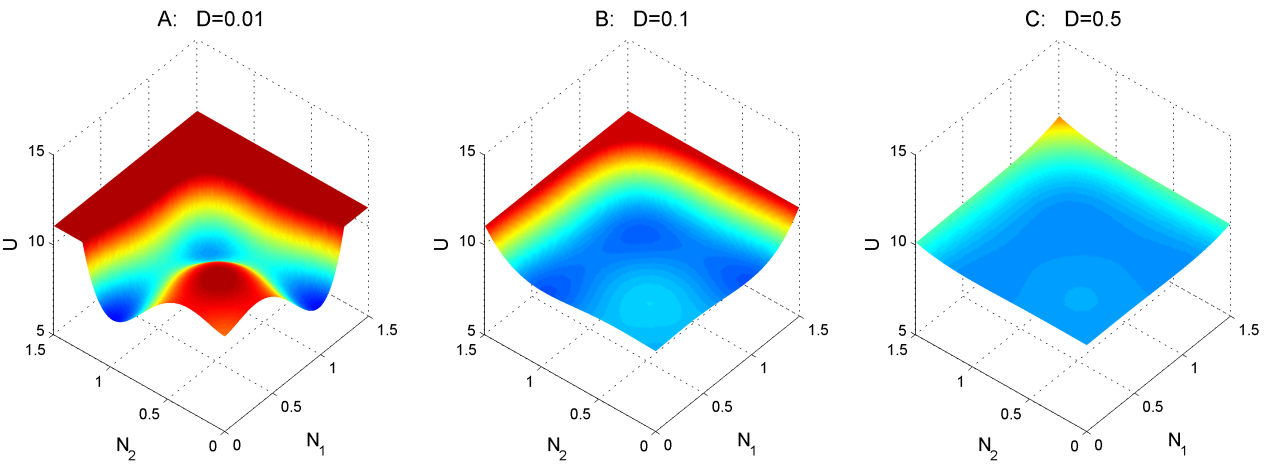


**Figure S6** The population potential landscapes for increasing parameter a_1_ of competition model.


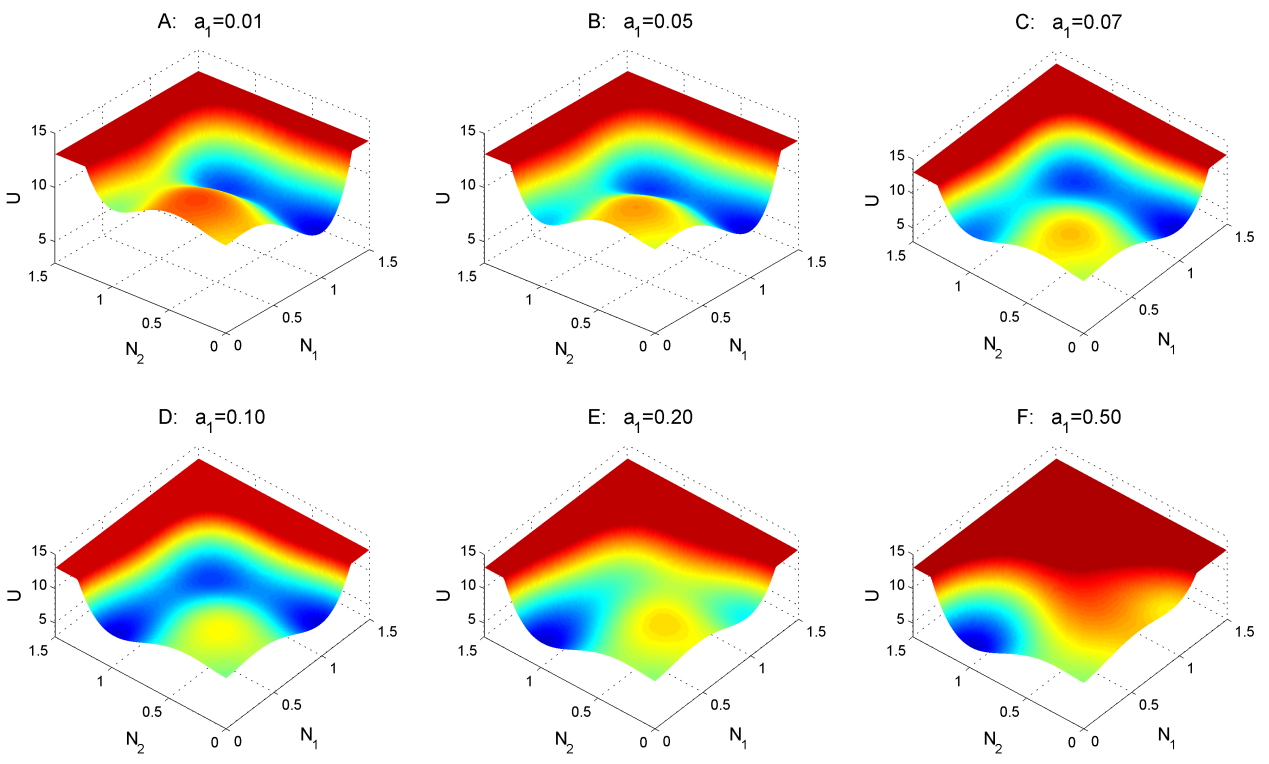


**Figure S7** The population potential landscapes for increasing parameter L_1_ of competition model.


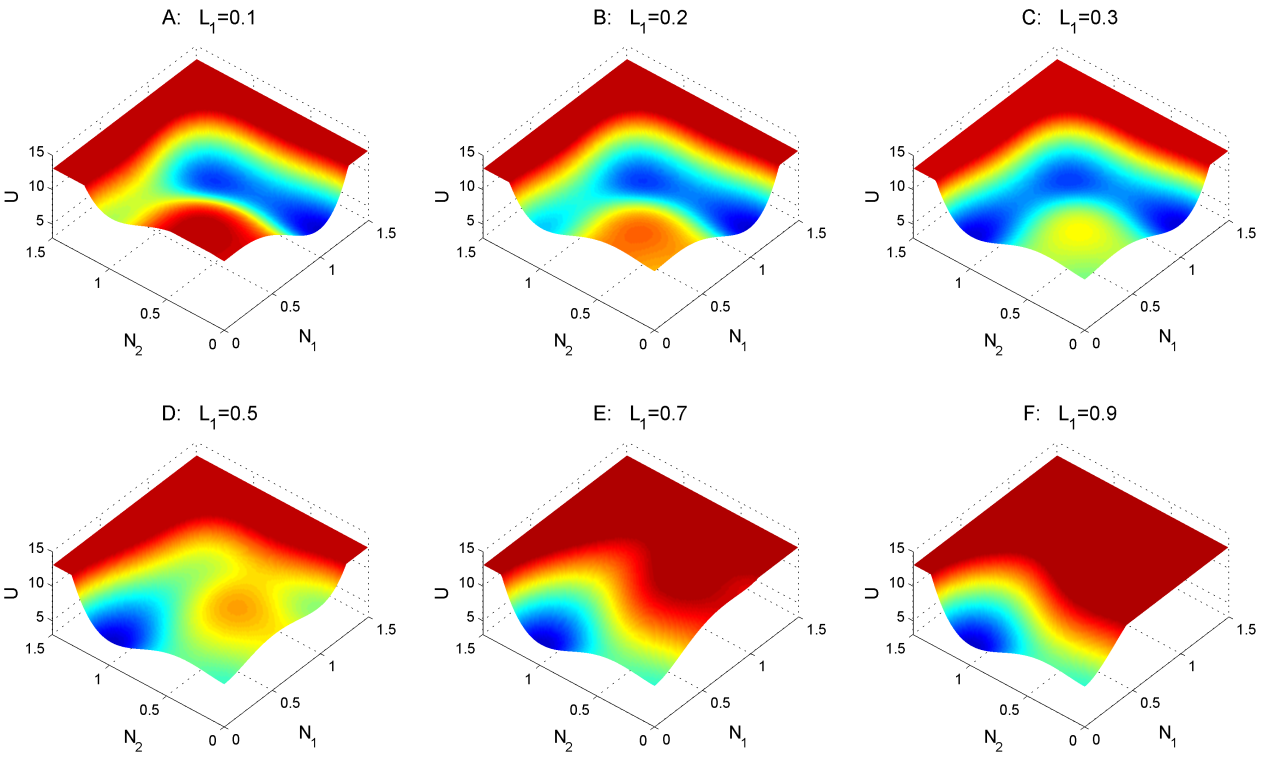


**Figure S8** The population potential landscapes for increasing parameter α of competition model.


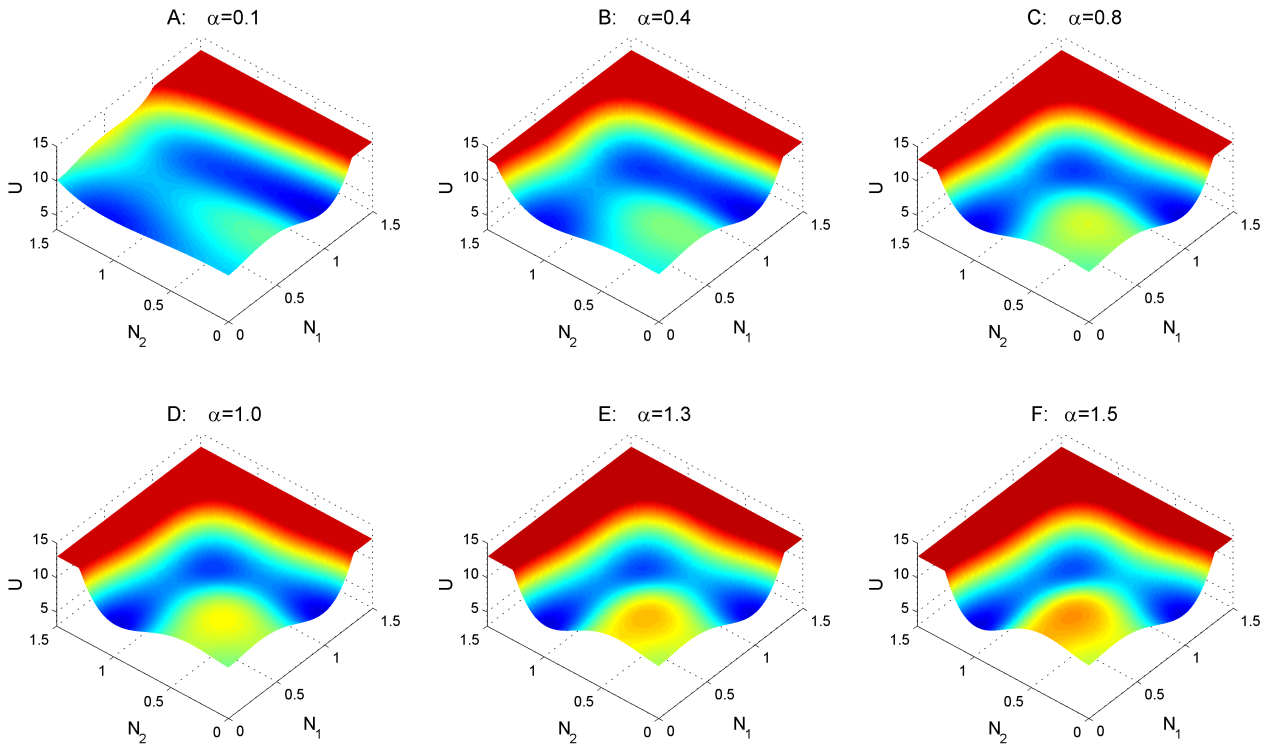


**Figure S9** The population potential landscapes for increasing diffusion coefficient D of mutualism model.


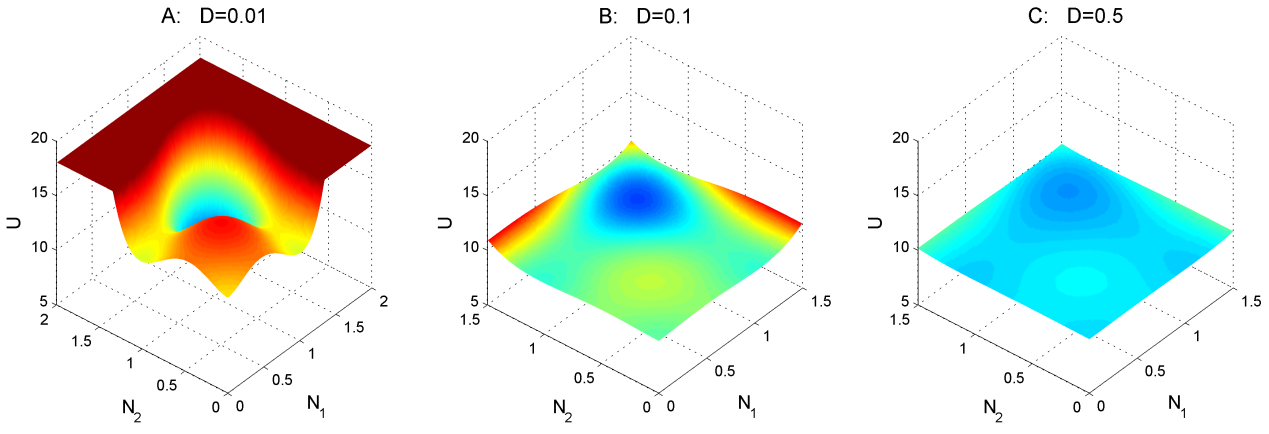


**Figure S10** The population potential landscapes for increasing parameter a_1_ of mutualism model.


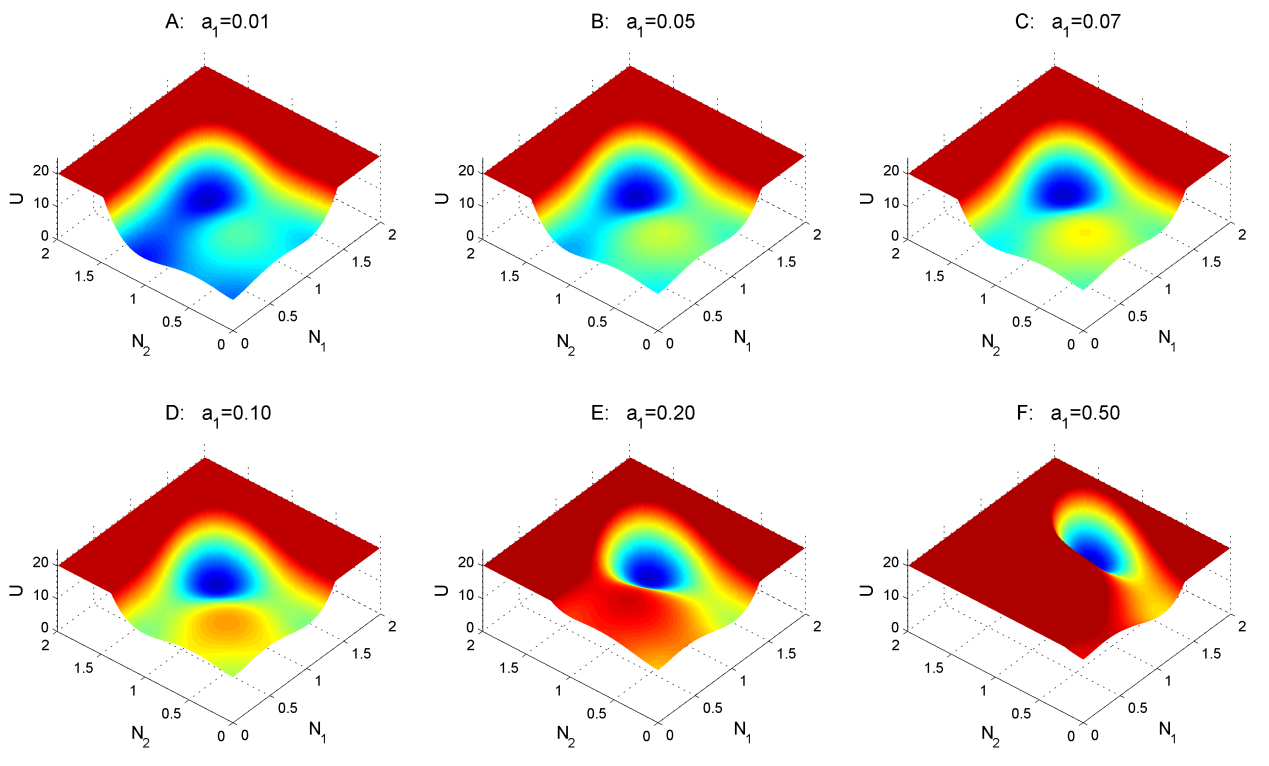


**Figure S11** The population potential landscapes for increasing parameter L_1_ of mutualism model.


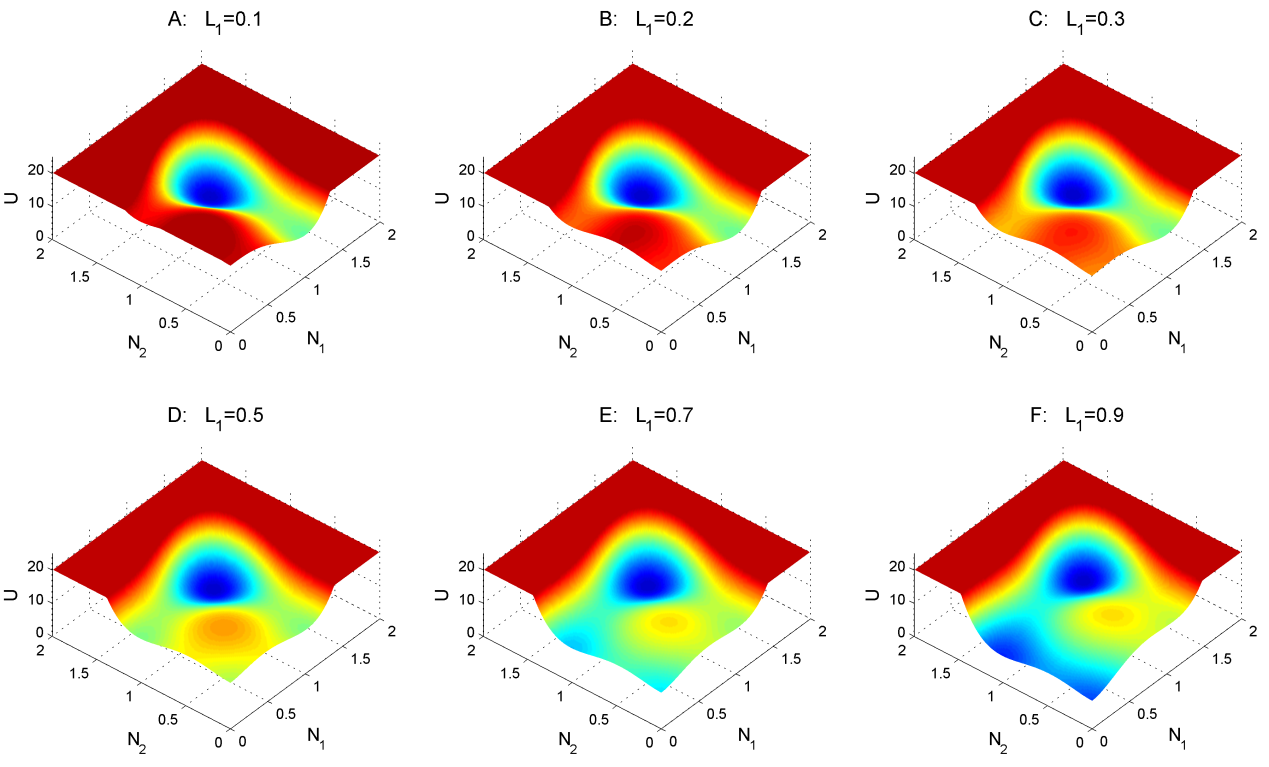


**Figure S12** The population potential landscapes for increasing parameter α of mutualism model.


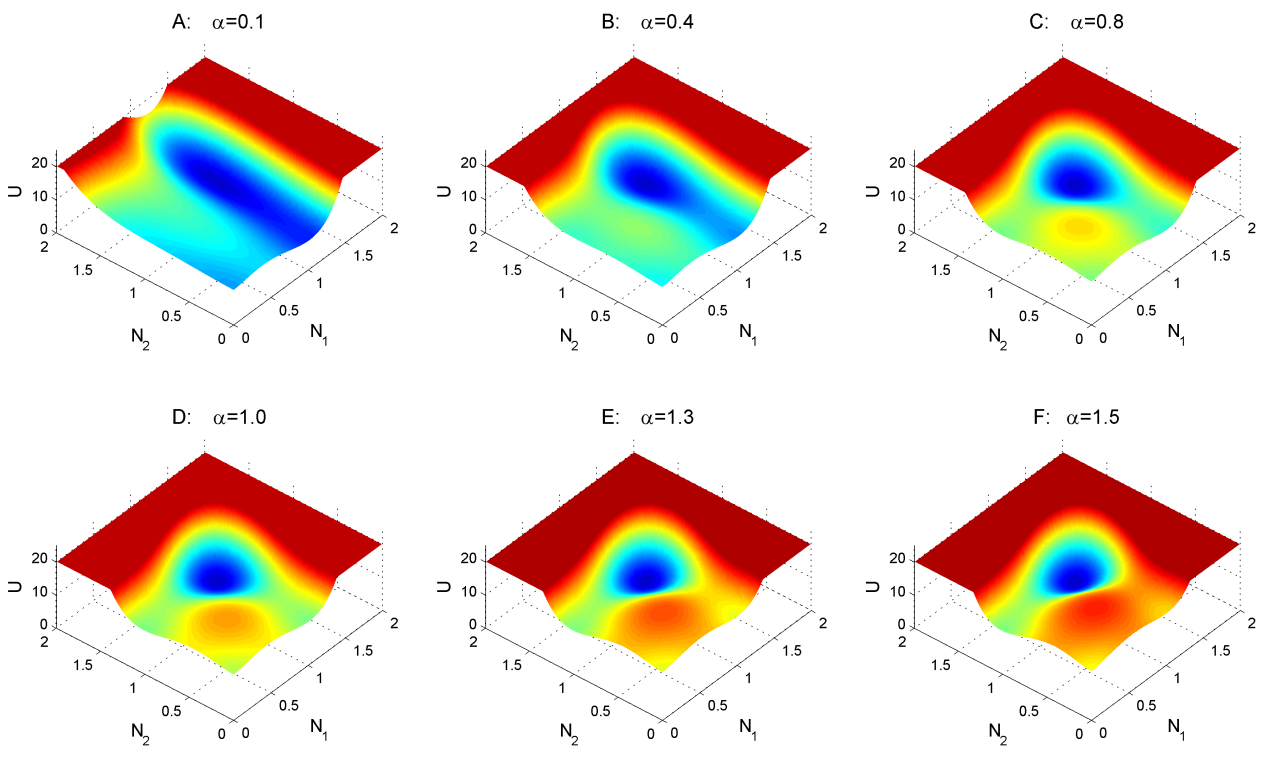

Supplement: File S1 — Supporting figures. Figure S1. The population potential landscapes for increasing diffusion coefficient of predation model. Figure S2. The population potential landscapes for increasing parameter of predation model.Figure S3. The population potential landscapes for increasing parameter of predation model. Figure S4. The population potential landscapes for increasing parameter of predation model. Figure S5. The population potential landscapes for increasing diffusion coefficient of competition model. Figure S6. The population potential landscapes for increasing parameter of competition model. Figure S7. The population potential landscapes for increasing parameter of competition model. Figure S8. The population potential landscapes for increasing parameter of competition model. Figure S9. The population potential landscapes for increasing diffusion coefficient of mutualism model. Figure S10. The population potential landscapes for increasing parameter of mutualism model. Figure S11. The population potential landscapes for increasing parameter of mutualism model. Figure S12. The population potential landscapes for increasing parameter of mutualism model. (DOC) [file pone.0086746.s001.doc]
